# New Species of *Rhamphomyia* Meigen (Diptera: Empididae) from the Palaearctic Region †

**DOI:** 10.3390/insects17040363

**Published:** 2026-03-26

**Authors:** Miroslav Barták, Igor Shamshev

**Affiliations:** 1 Department of Zoology and Fisheries, Faculty of Agrobiology, Food and Natural Resources, Czech University of Life Sciences Prague, Kamýcká 129, CZ-165 00 Praha-Suchdol, Czech Republic; 2 Laboratory of Insect Systematics, Zoological Institute of Russian Academy of Sciences, Universitetskaja nab. 1, St. Petersburg 199034, Russia; shamshev@mail.ru; 3 Laboratory of Agricultural Entomology, All-Russian Institute of Plant Protection, Podbelskogo Rd, 3, Pushkin, St. Petersburg 196608, Russia

**Keywords:** Empidoidea, dance flies, new species, China, Kazakhstan, Mongolia, Russia, Slovakia, Spain, Turkmenistan

## Abstract

Twenty-two new species of the genus *Rhamphomyia* Meigen (Diptera: Empididae) are described and illustrated from different parts of the Palaearctic region (mostly from East Asia): *Rhamphomyia* (*Calorhamphomyia*) *iridescens* sp. nov. (Mongolia, Russia); *R*. (*Pararhamphomyia*) *acuticauda* sp. nov. (Slovakia); *R*. (*P*.) *amurensis* sp. nov. (Russia); *R*. (*P*.) *angustitibia* sp. nov. (Russia); *R*. (*P*.) *basitarsata* sp. nov. (China, Russia); *R*. (*P*.) *bifurcata* sp. nov. (Russia); *R*. (*P*.) *epandriata* sp. nov. (Russia); *R*. (*P*.) *globulicauda* sp. nov. (Russia); *R*. (*P*.) *haladai* sp. nov. (Kazakhstan); *R*. (*P*.) *indigirka* sp. nov. (Russia); *R*. (*P*.) *krivosheinae* sp. nov. (Russia); *R*. (*P*.) *morgunovka* sp. nov. (Turkmenistan); *R*. (*P*.) *norgensis* sp. nov. (Norway, Russia); *R*. (*P*.) *nudifemorata* sp. nov. (Russia); *R*. (*P*.) *plutenkoi* sp. nov. (Russia); *R*. (*P*.) *sausai* sp. nov. (China); *R*. (*P*.) *schachti* sp. nov. (Spain); *R*. (*P*.) *seticauda* sp. nov. (Russia); *R*. (*P*.) *spinicauda* sp. nov. (Russia); *R*. (*P*.) *spiraliseta* sp. nov. (Russia); *R*. (*P*.) *subcurvitibia* sp. nov. (Russia); *R*. (*P*.) *zeegersi* sp. nov. (Russia).

## Simple Summary

This paper aims to contribute to the knowledge of Diptera biodiversity in the Palaearctic region. The genus *Rhamphomyia* (Empididae, Empidinae) is a large genus, comprising approximately 600 described species worldwide. Owing great species diversity, *Rhamphomyia* is a marked element of the biota of many biotopes. Here, we provide descriptions of twenty-two species of *Rhamphomyia* new to science from different parts of the Palaearctic region (mostly from East Asia). Characters important for morphological identification are discussed and illustrated.

## 1. Introduction

The Empididae, or dance flies, are small to medium-sized (body length 1–10 mm, but usually about 5 mm) dark or greyish flies (rarely yellow or metallic green). The group is one of the largest families of the Diptera superfamily Empidoidea and has worldwide distribution (except Antarctica) comprising about 3500 described species [1]. Many representatives of empidids are readily recognisable in nature due to their peculiar swarming behaviour and due to the presence of a relatively long proboscis [2].

The genus *Rhamphomyia* Meigen, 1822 belongs to the empidid subfamily Empidinae and the tribe Empidini [2]. *Rhamphomyia* is one of three cosmopolitan mega-genera of Empididae, alongside *Empis* Linnaeus and *Hilara* Meigen, that demonstrate a great morphological diversity of the group. Currently, about 600 described species of *Rhamphomyia* are known worldwide [1,3,4,5]. However, about the same number of species remains unnamed, particularly in North America and the eastern part of Eurasia.

The males of many species of *Rhamphomyia* exhibit curious modifications of the legs and postabdomen (sometimes bilaterally asymmetrical). The females often possess attractive ornamentations including pennate legs, enlarged, darkened, or maculated wings, etc. Adults of *Rhamphomyia* demonstrate a great ethological diversity. They form swarms, except for species of the subgenus *Lundstroemiella* Frey, which transferred their mating activity to ground vegetation [6,7]. Males capture animal prey, which they transfer to the female as “wedding present” (except for species of the subgenera *Lundstroemiella* and *Holoclera* Schiner). The presence of ornamentations in females of many species of *Rhamphomyia* is explained by probable sex role-reversed mate selection in these flies [8]. Owing great species diversity, *Rhamphomyia* is a marked element of the biota of many biotopes. Adults of several species are important pollinators in high mountains and in boreal environments [9].

It is evident that an enormously large number of described species requires a subdivision of the genus into some recognisable units. Saigusa [4] has provided a historical review of *Rhamphomyia* classification. Up to now, 18 subgenera of *Rhamphomyia* have been proposed by different authors (12 subgeneric names refer to the Holarctic and 2 names to the Neotropical realms). The Holarctic subgenera *Rhamphomyia* (*Rhamphomyia*) and *R*. (*Pararhamphomyia* Frey) are the most diverse groups, including more than 90% of known species of the genus. However, a comprehensive subgeneric classification of *Rhamphomyia* remains elusive. The main difficulties in *Rhamphomyia* subgeneric classification are the following: very high morphological diversity; weak phylogenetic base-ground; incongruence between male and female units; ranking in classification [4,10]. According to our unpublished data, almost 50 species groups could be identified only in the Palaearctic fauna of *Rhamphomyia* and maybe even more in the Nearctic fauna. It appears that *Rhamphomyia* represents an artificial assemblage of unrelated species and groups of species, some being closely related to *Empis* s. str. (e.g., *Aclonempis* and some species of *Pararhamphomyia*) or even to other subgenera of *Empis* and some deserving generic status (e.g., *Amydroneura* Collin, *Lundstroemiella* Frey, *Holoclera nigripennis*-group). Traditionally, the absence of wing vein R_4_ in *Rhamphomyia* distinguishes it from *Empis*. However, it has been shown that *Empis* lost vein R_4_ several times [11,12].

*Rhamphomyia* is primarily a Holarctic group, where it is particularly diverse in temperate and boreal regions [13,14,15,16]. In the Palaearctic, the genus was most extensively studied in Europe and some of the closest regions [17,18,19,20,21]. The key to *Rhamphomyia* species of Europe was provided by Barták [10], of the Middle East by Barták et al. [18]. Many species from Asia have been keyed by Barták [22], Sinclair et al. [13] and Shamshev et al. [15].

However, many new species of *Rhamphomyia* are still waiting description. The lack of the taxonomic data that prevents an understanding of individual species is a prerequisite for estimating their functional value with regard to the ecosystem services they provide. Our paper includes descriptions and illustrations of 22 new species of the genus *Rhamphomyia,* which were collected from different parts of the Palaearctic region (mostly from East Asia).

## 2. Materials and Methods

This study is based on material deposited in the Czech University of Life Sciences, Prague (CULSP), Museum of Zoology, Lund University, Lund, Sweden (MZLU); Siberian Zoological Museum, Institute of Systematics and Ecology of Animals of the Russian Academy of Sciences, Novosibirsk (SZM), the Zoological Museum of Moscow University, Moscow (ZMMU) and the Zoological Institute, Russian Academy of Sciences, St. Petersburg, Russia (ZISP).

Label data for primary types are cited in full with original spelling, punctuation and date. Label lines are delimited by a slash (/), and the data from each label are separated by two slashes (//). The repository of specimens is given in parentheses. Additional information to label data (coordinates, translation to English, etc.) is included in square “[ ]” brackets. Secondary type data and additional material are abridged.

Pinned specimens were used to take photographs using a Canon EOS 11 40D camera (Canon Inc., Tokyo, Japan) with a Canon MP-E 65 mm objective and Nikon SMZ 1500 (Nikon Inc., Tokyo, Japan) stereomicroscope equipped with a Canon EOS 700D digital camera, with multiple layers combined using the Helicon Focus software (Helicon Soft Ltd., Kharkiv, Ukraine). Images served as models for hand drawings, and details were added by directly observing the dissected genitalia (except noted). To facilitate observations, the terminalia were macerated during 24 h in cold 10% KOH then immersed for 5–10 min in 85% lactic acid or 8% acetic acid and viewed in glycerine. Terms used for adult structures mostly follow those of Cumming & Wood [23]. The body measurements of setae length were taken from dry specimens by means of an ocular micrometre on a Nikon SMZ 1500 binocular microscope.

Ratio of antennal segments = length of scape: pedicel: postpedicel: style (in 0.01 mm). Male body length was measured from antennal base to the tip of last abdominal segment (without the genitalia) and female body length from the base of antennae to the tip of the cerci. Wing measurements: M_2_/dm = length of vein M_2_: greatest length of discal medial cell (=cell dm); M_3_/Db—M_4_ ap/mp—M_4_ apical portion/middle portion; wl/ww = wing greatest length (from basicosta to apex): wing greatest width. Length of frons is measured from front margin of front ocellus to antennal base. Thoracic setae are counted on one side of the body (except scutellars).

The following abbreviations are used for administrative units of the Russian Federation in type of material and distribution sections (arranged alphabetically): AO—Autonomous Okrug, District (=rayon), Prov.—Province (=oblast), Rep.—Republic, Terr.—Territory (=kray). Transliteration of Cyrillic (Russian) names follows those of Shamshev [14]. The taxa are arranged hierarchically and alphabetically according to the categories of subgenus, and species.

## 3. Taxonomy

The new species described below belong to two subgenera of *Rhamphomyia*, namely *R*. (*Calorhamphomyia* Saigusa) and *R*. (*Pararhamphomyia* Frey). The subgenus *R*. (*Calorhamphomyia*), with type species *R*. (*E*.) *latistriata* Frey, is a relatively small group comprising 23 species (including a species described herein) [1]. The subgenus is mostly known from East Asia, but two species were also recognised from North America. The subgenus *R*. (*Pararhamphomyia*) is one of the two largest groups of the genus, alongside *R*. (*Rhamphomyia*), comprising about 170 species (including new species described herein). The majority of *R*. (*Pararhamphomyia*) species is known from the Holarctic. An arrangement of a new species of *R*. (*Pararhamphomyia*) in a species group is discussed under the section Differential Diagnosis. A catalogue of described species is provided after the descriptions.

### 3.1. Rhamphomyia (Calorhamphomyia) iridescens sp. nov.

Zoobank link: urn:lsid:zoobank.org:act:7CFE5F6D-2C33-4F3D-94EA-9533D109C926

(Figure 1A–D)

**Type material: HOLOTYPE ♂**, labelled: RUSSIA: Altai Terr./Krasnoschekovskiy Distr./Tigirek vill., 51°08′ N, 83°03′ E/550 m, 9–12.vii.2014, leg. I. Shamshev (ZISP, INS_DIP_0000880). **PARATYPES: MONGOLIA:** Selenginskiy aimak, 30 km ENE Dzun-Khara, 6.viii.1975, Nartshuk (1 ♀, ZISP). **RUSSIA. Altayskiy Terr.:** same data as holotype (2 ♂, 5 ♀, ZISP). **Altay Rep.:** Onguday env. 50.77 N 86.09 E, 840 m, 8–13.vii.2016, N. Vikhrev (1 ♀, ZMMU). **Amurskaya Prov.:** Zeja, 15.viii.1982, leg. A. Shatalkin (1 ♂, CULSP). **Buryatia Rep.:** Mondy, Verkhnie Sayany, 1400 m, birch forest, 24.vii.1965, Gorodkov (31 ♂, 4 ♀, ZISP); Kyren, Verkhnie Sayany, 700 m, 6 km E Khorgun, sedge swamp above the water of a stream, 11.vii.1965, Gorodkov (3 ♂, ZISP). **Krasnoyarskiy Terr.:** Vyeszhiy log, 2.vii.1912, Tugarinova (1 ♀, ZISP). **Tuva Rep.:** Uyuk River, 800 m, 52.07 N 94.04 E, 1–3.vii.2017, N. Vikhrev (5 ♂, 2 ♀, ZMMU). **Zabaykalskiy Terr.:** Baley, 18.vii.1971, V. Richter (1 ♂, ZISP); 15 km SE Chita, river Nikishikha, 28.vii.1975, Kasparyan (1 ♂, ZISP); Chita, 21.vii.1976, V. Richter (1 ♂, ZISP); railway station Ingoda, floodplain of river Ingoda, 7.vii.1971, V. Richter (2 ♂, 3 ♀, ZISP).

**Diagnosis:** Medium-sized species (body 5–5.5 mm) of *Rhamphomyia* with biserial acrostichal setae, setose prosternum, partly pale setose body and extensively yellow legs. Male: hind basitarsus slender; abdominal tergite 7 with transversal dorsal “bump” and large, broadly rounded, lateral, finger-like process on each side. Female: wing somewhat narrower in proximal 1/3 than in distal 1/3, cell dm short, anal vein (CuA+CuP) incomplete.

**Etymology:** The name of the species stresses conspicuously iridescent wing.

**Figure 1:**
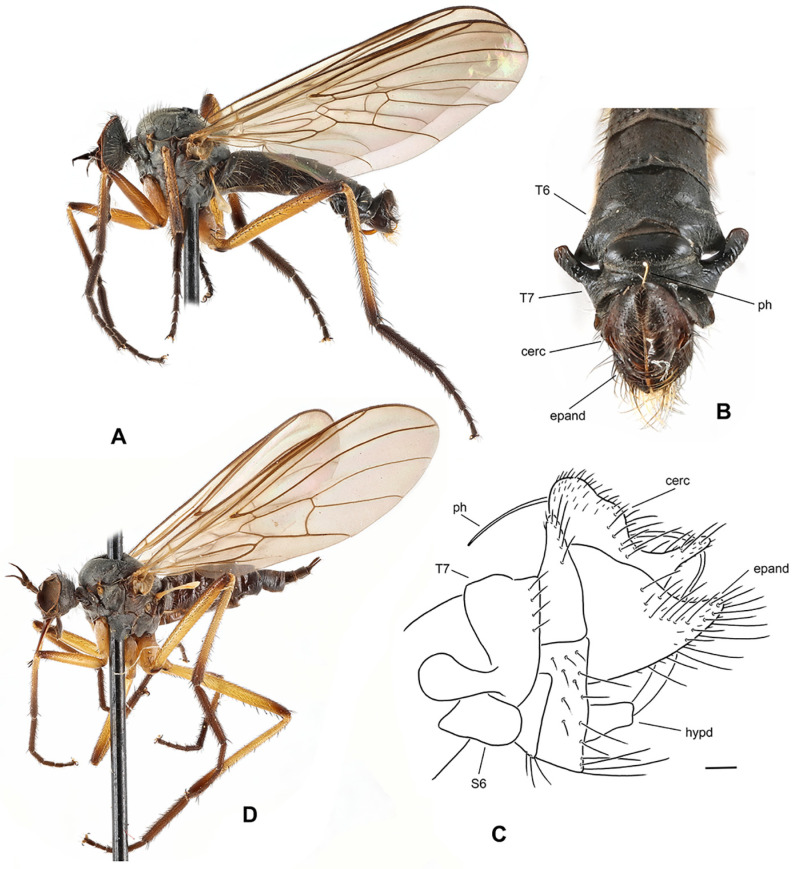
*Rhamphomyia* (*Calorhamphomyia*) *iridescens* sp. nov. (**A**) = Male holotype, habitus, lateral view. (**B**) = Male postabdomen, dorsal view. (**C**) = Male terminalia, lateral view. (**D**) = Female habitus, lateral view. Abbreviations: cerc—cercus, epand—epandrial lamella, hypd—hypandrium, ph—phallus, S—sternite, T—tergite. Scale bar 0.1 mm.

**Description: Male** (Figure 1A). Length: body 4.9–5.3 mm (without genitalia), wing 5.5–6.4 mm. **Head** black, mostly light grey microtrichose. Eyes holoptic, upper ommatidia slightly enlarged. Frons bare. Ocellar setae black, half as long as frons, ocellar triangle with 2 pairs of additional setae 1/2–1/3 as long as ocellars. Face about 0.21 mm broad ventrally and 0.27 mm long, bare. Occiput covered with sparse black setae dorsally, postocular row present only in dorsalmost part; postgena with dense, fine, pale setae. Antenna black, scape and pedicel slightly paler than postpedicel, rather short setose (longest setae about 0.15 mm long); antennomere ratio = 15:9:36:15. Labrum brownish, shiny, slightly shorter than head height. Palpus brown, short, bearing a few setae. Gena narrow, microtrichose; clypeus microtrichose. **Thorax** brownish black, light grey microtrichose, in dorsal view with indistinct dark vittae under rows of acrostichal and dorsocentral setae (more distinct in posterior view). Chaetotaxy: proepisternum with about 20 pale setae on lower part, proepisternal depression bare (upper part of proepisternum); prosternum with 4–5 long pale setae on each side; postpronotal lobe with 1 strong, black and numerous additional, much shorter, fine, pale setae; acrostichals dark, 1–2-serial, fine, short, sparse, lacking on prescutellar depression; about 24 irregularly biserial, very fine, dark, short dorsocentrals (about 0.15 mm long in middle of rows), 1–2 prescutellars; 0–1 barely distinguishable presutural intra-alar; 1 presutural supra-alar (and several additional dark or pale setae outside dorsocentrals); 2 notopleurals (anterior part of notopleuron bare or with 1–3 fine pale setae); 1–2 postsutural supra-alar(s) (sometimes with 1–2 additional fine setae); 1 postalar; 2 long and 2 much shorter scutellar setae; laterotergite with numerous pale setae. **Legs** colour: all femora yellow (except brownish apex); fore tibia yellow at about basal 1/3, becoming brown toward apex; mid and hind tibia yellow, narrowly brownish at apex; tarsi entirely blackish brown. Legs microtrichose (hind femora subshiny), covered with brown setae; all femora whitish tomentose ventrally. Coxae yellow, microtrichose, covered with strong dark and fine pale setae. Fore femur with short setae dorsally, bare ventrally. Fore tibia with uniform, moderately long, posterodorsal setae (slightly longer than tibia width), ventral setae short, dense and fine. Mid femur with row of short, spinose, anteroventral setae (about 1/3 as long as femur width), similar row of longer spinose posteroventral setae (slightly shorter than femur width) and short setae dorsally. Mid tibia with two rows of spinose ventral setae (nearly as long as tibia width) and with 4–5 pairs of setae dorsally (longest preapical anterodorsal seta about 3× as long as tibia width. Hind femur bare on ventral half (except a few preapical setae), dorsal setae short, 1 row of anterodorsal setae present. Hind tibia with 4–5 pairs of rather short dorsal setae (at most slightly longer than tibia width); 1 rather long seta in posteroapical comb. Basitarsi of all legs slender, short setose; in addition, mid and hind basitarsi with short ventral spines. **Wing** clear, iridescent; stigma brown, narrow; veins brown, anal vein (CuA+CuP) complete (usually with short portion beyond middle weakened). Costal seta absent, axillary excision very acute (40°). Measurements: M_2_/dm = 1.1–1.2, M_4_ ap/mp = 1.2, wl/ww = 3.3–3.4. Halter pale yellow; calypter yellow, pale fringed. **Abdomen** blackish brown; mostly faintly microtrichose, dorsal “hump” and lateral processes on tergite 7 as well as sternite 6 shiny. Chaetotaxy: covered with pale to yellow setae; hind marginal setae on sides of tergites 2–3 subequally long as corresponding segment, remaining segments much shorter setose, discal setae slightly shorter than marginals; dorsum of tergites short setose. Pregenital segments 6–8 modified (Figure 1B): segment 6 with fused tergite and sternite, sternite 6 deeply concave posteriorly; tergite 7 with transversal dorsal “bump” and large, broadly rounded, lateral, finger-like process on each side. **Terminalia** as in Figure 1C (not macerated). Cerci, epandrium and hypandrium brownish, phallus yellow; cerci covered with black setae; epandrial lamella with dense, relatively long, mostly yellow setae apically. Epandrial lamella subtriangular; cercus deeply concave dorsally; phallus forms simple bow with shallow outgoing loop in about middle.

**Female** (Figure 1D). Length: body 5.3–5.5 mm, wing 6.1–6.4 mm. Similar to male but with the following exceptions. Eyes broadly dichoptic; frons nearly parallel-sided and nearly as broad as face, bearing minute marginal setulae. Occiput with somewhat shorter and sparser setae than in male. Thorax with generally shorter setae than in male. Legs colour pattern as in male, only with simple setae. Mid femur with rows of anteroventral and posteroventral spine-like setae becoming shorter toward base (longest setae nearly as long as femur width at middle); mid tibia bearing 3–4 anterodorsal and 3–4 posterodorsal short setae (position and robustness variable), with hardly prominent spinule-like setae ventrally. Hind femur with short setae anterodorsally (except 2–3 subapicals); hind tibia with short setae dorsally. Wing modified, almost twice narrower in proximal 1/3 than in distal 1/3, anal lobe small, axillary excision nearly 90°, vein CuA+CuP usually incomplete (sometimes as in male); cell dm short, with apex only slightly elongate, apical portion of M_4_ slightly shorter than its middle portion. Abdomen brownish, mostly faintly greyish microtrichose (slightly denser on segments 6–8), with short, fine, mostly pale setae (longer on tergites 1–3 laterally); cercus long, slender, covered with short, dark setulae.

**Differential diagnosis:** *Rhamphomyia iridescens* sp. nov. belongs to the subgenus *Calorhamphomyia* Saigusa in broad concept gathering not only “typical” representatives (with absent prealar setae and shiny clypeus) but also other species sharing the wing with an acutely incised axillary angle, complete vein CuA+CuP and well-developed lateral projections on tergite 7 [24]. The new species can be readily distinguished from other Palaearctic *Rhamphomyia* (except the most allied *R. insignis* Loew) by the combination of biserial acrostichal setae, setose prosternum, partly pale setose body and yellow legs. *Rhamphomyia insignis* has postpedicel at least 5 times as long as style, the lateral projection on the tergite 7 of the male is pointed, rather horn-like, the hind femur bears short, black, ventral spines and the hind tibia is long setose dorsally. Moreover, there are clear differences in male genitalia. The female of the new species differs from the female of *R*. *insignis* by short discal cell, incomplete vein CuA+CuP and some other characters.

**Distribution:** Mongolia, Russia (Altay Rep., Altayskiy Terr., Amurskaya Prov., Buryatia Rep., Krasnoyarskiy Terr., Tuva Rep., Zabaykalskiy Terr.).

**Dates of occurrence:** July–August.

### 3.2. Rhamphomyia (Pararhamphomyia) acuticauda sp. nov.

Zoobank link: urn:lsid:zoobank.org:act: 4222FF3E-6706-46CE-9680-97183E22D0CE

(Figure 2A–C)

**Type material: HOLOTYPE ♂**, labelled: Slovakia mer., Kamenín, salty meadow, 49°53′ N, 18°39′ E, 130 m, 25.iv.1986, leg. M. Barták (CZU). **PARATYPE. SLOVAKIA:** same data as holotype (1 ♂, CULSP).

**Diagnosis:** Rather small species of *Rhamphomyia* (*Pararhamphomyia*) (body about 3 mm) with black setose body, light grey mesoscutum, uniserial dorsocentral setae, dark legs and pale halteres. Male: legs simple; epandrial lamella long, very narrow, subtriangular, pointed at apex, without dorsal setose hump; phallus forming only one loop.

**Etymology:** The epithet refers to the sharply tipped epandrial lamellae.

**Figure 2:**
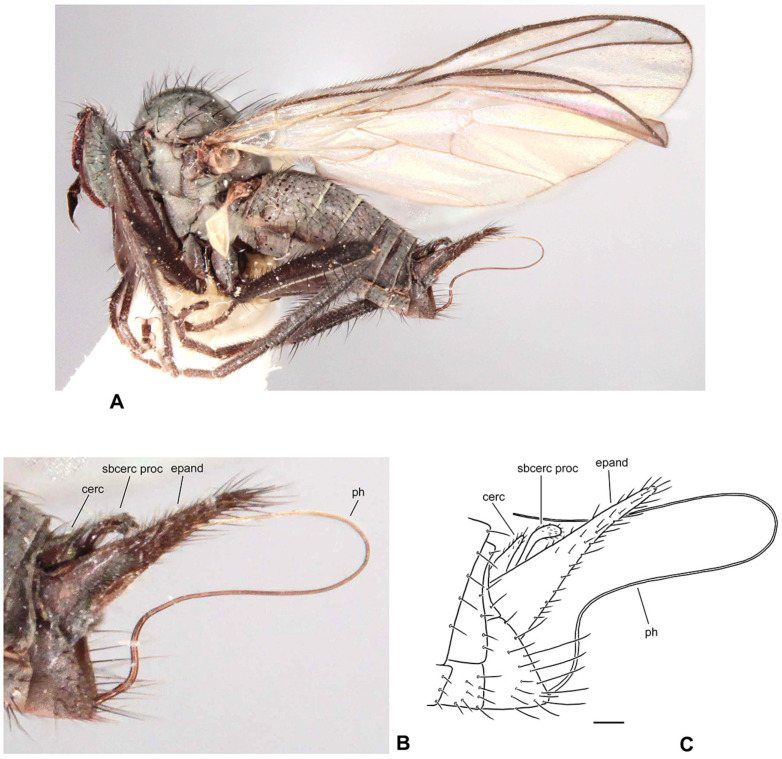
*Rhamphomyia* (*Pararhamphomyia*) *acuticauda* sp. nov., male holotype. (**A**) = Habitus, lateral view. (**B**) = Terminalia, lateral view. (**C**) = Outline of terminalia, lateral view. Abbreviations: cerc—cercus, epand—epandrial lamella, ph—phallus, sbcerc proc—subcercal process. Scale bar 0.1 mm.

**Description: Male** (Figure 2A). Length: body 3.0 mm (without hypopygium), wing 3.4 mm. **Head** black, black setose, head regions mostly grey microtrichose. Eyes holoptic; upper ommatidia enlarged. Frons bare. Ocellar setae half as long as frons; posterior part of ocellar triangle with 1–2 pairs of additional short setae. Face light grey microtrichose, about 0.16 mm broad ventrally and 0.10 mm long, bare; clypeus microtrichose. Occiput with rather spars, moderately long setae; postocular row present only in dorsal part of occiput. Antenna black, scape and pedicel rather short setose (longest setae about 0.08 mm long); antennomere ratio = 9:7:29:10. Labrum brown, shiny, 2/3 as long as head height. Palpus brown, short, bearing a few short setae. Gena narrow, microtrichose. **Thorax** black, light grey microtrichose, black setose; mesoscutum without distinct vittae. Chaetotaxy: proepisternum with 2 setae on lower part and bare on upper part in front of spiracle (=proepisternal depression); prosternum bare; postpronotal lobe with 1 long and 3–6 shorter setae; acrostichals narrowly biserial, about 12–14 per row; dorsocentrals uniserial, 7–9 per row (sometimes with 2 additional anterior setae standing outside row), rather sparse, slightly longer than acrostichals (0.25 mm in middle of row), ending in 1–2 prescutellars; 1 presutural intra-alar (with 0–1 additional seta), 1 presutural supra-alar (with 0–1 additional seta), 3 notopleurals (lower seta situated unusually cranially (with 1–3 additional, rather long setae on anterior part of notopleuron), 2 postsutural supra-alars (with 2–3 additional setae on prealar area), 1 postalar, 2 long and 2 shorter (half as long) scutellars; laterotergite with numerous setae. **Legs**, including coxae, brownish black, microtrichose, black setose. Fore femur with complete rows of anteroventral and posteroventral setae (nearly as long as femur width). Fore tibia with almost uniform setation dorsally (nearly as long as tibia width), ventral setae half as long. Mid femur with irregular row of anteroventral setae (about as long as femur width); posteroventrals longer, in apical third nearly twice as long. Mid tibia with 3 long anterodorsal setae, including preapical seta (nearly 3× longer than tibia width); posterodorsal setae shorter and fine (besides 1 seta of preapical circlet), 2 almost regular rows of anteroventral and posteroventral setae (shorter than tibia width, 1–2 posteroventrals sometimes more distinct). Hind femur with 2 rows of 6–8 strong anteroventral and posteroventral setae in apical third (nearly twice as long as femur width), anteroventrals in basal half of femur short, posteroventrals slightly longer, dorsal setae short. Hind tibia with 3–4 anterodorsals (nearly 1.5× as long as tibia width); posterodorsal setae less differentiated, ventral setae nearly as long as tibia width; 1 long seta in posteroapical comb. Basitarsus of all legs slender, short setose, with short, ventral, spine-like setae. **Wing** membrane clear, pterostigma yellowish; veins pale yellowish-brown, CuA+CuP weakened in basal half, reaching wing margin apically. Basal costal seta present. Axillary excision 90º. Measurements: M_2_/dm = 1.2, M_4_ ap/mp = 1.9, wl/ww = 2.8. Calypter yellow, with dark fringe. Halter yellow. **Abdomen** black, densely light grey microtrichose. Chaetotaxy: covered with only dark setae; hind marginal setae on sides of tergites 2–4 nearly as long as corresponding segment, on segments 5–6 shorter, discal setae subequal to marginals; dorsum of tergites shorter setose. **Terminalia** as in Figure 2B (not macerated); cerci and epandrium concolourous with abdominal tergites, greyish microtrichose; epandrial lamella long, subtriangular (lateral view), sharply tipped, without tufts of setae, without preapical swellings; hypandrium invisible in situ; cercus subtriangular, pointed at apex; “subcercal process” elongate.

**Female.** Unknown.

**Differential diagnosis:** *Rhamphomyia* (*P.*) *acuticauda* sp. nov. belongs to the species rich group of *Pararhamphomyia* with uniserial dorsocentral setae, dark legs, pale halters and entirely black setose body. The new species differs from other species of this group by having simple legs, the phallus not forming several loops, the mesoscutum light grey microtrichose and very characteristic shape of the terminalia. An elongate epandrium equally narrowing apically without dorsal setose hump is really exceptional. Because dorsocentral setae have tendency to be biserial, this new species may be wrongly arranged in the group of *Pararhamphomyia* with biserial dorsocentrals, where it resembles (except terminalia) *R. hoeli* Frey. In addition, several species with partly yellow legs belong to the same group of species. The female remains unknown.

**Distribution:** Slovak Republic.

**Dates of occurrence:** April.

### 3.3. Rhamphomyia (Pararhamphomyia) amurensis sp. nov.

Zoobank link: urn:lsid:zoobank.org:act: AB0C4525-C3F7-4354-988E-7A8E4F23B750

(Figure 3A,B)

**Type material: HOLOTYPE** ♂, labelled: [in Cyrillic, **RUSSIA**], Amursk. obl. [=Amurslaya oblast’, province]/g. [=gorod, town] Zeja [53°44′ N 127°15′ E], 6.vi.1978/A. Shatalkin (ZMMU). **PARATYPES: RUSSIA. Amurskaya Prov.:** same data as holotype (2 ♂, 1 ♀, CULSP); same locality, 7.vi.1978, A. Shatalkin (1 ♂, ZMMU); same locality, 15.vi.1978, A. Shatalkin (1 ♂, ZMMU); same locality, 6.vi.1978, A. Shatalkin (1 ♀, ZMMU); same locality, 16.vi.1982, M. Krivosheina (2 ♂, 1 ♀, ZISP). **Yakutia:** 7 km N Yakokit, 58.95 N 125.85 E, meadow, 24–27.vi.2022, O. Kosterin (1 ♂, ZMMU).

**Diagnosis:** Medium-sized species (body 4–5 mm) of the *R.* (*P.*) *tipularia* group; antenna with scape and pedicel yellow, dorsocentral setae uniserial calypter brown fringed. Male: cercus elongated, nearly parallel-sided, with small subbasal projection ventrally; “subcercal” process long and bifurcated at apex; epandrial lamella subtriangular, narrow, ending in yellow shiny hook with black spine at tip; phallus bent before apex.

**Etymology:** The species is named after the type locality.

**Figure 3:**
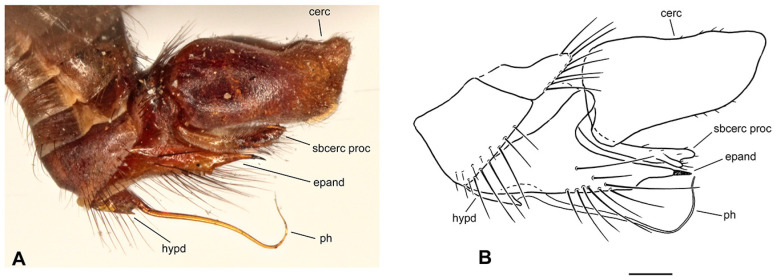
*Rhamphomyia* (*Pararhamphomyia*) *amurensis* sp. nov., male terminalia. (**A**) = Lateral view. (**B**) = Outline. Abbreviations: cerc—cercus, epand—epandrial lamella, hypd—hypandrium, ph—phallus, sbcerc proc—subcercal process. Scale bar 0.1 mm.

**Description: Male.** Length: body 4.0–4.7 mm (without genitalia), wing 5.9–6.6 mm. Head black, black setose, head regions mostly light grey microtrichose. Eyes holoptic, upper ommatidia enlarged. Frons bare. Ocellar setae very short (only 0.20 mm), ocellar triangle with several additional short setae in posterior part. Face about 0.19 mm broad ventrally and 0.20 mm long, bare. Occiput sparse short setose, all occipital setae confined to two rows in dorsal 2/3. Antenna with scape and pedicel yellow, short setose, postpedicel and stylus black; antennomere ratio = 10:9:36–42:10. Labrum yellowish brown, shiny, slightly shorter than head height. Palpus brown, short, bearing a few setae. Gena narrow, microtrichose; clypeus microtrichose. **Thorax** brownish black but prothorax sclerites (including postpronotal lobe) and laterotergite yellowish translucent; entirely light grey microtrichose, mesoscutum with scarcely distinct, somewhat darker vittae between rows of acrostichal and dorsocentral setae. All setae black to brown. Chaetotaxy: proepisternum with 1 seta on lower part, bare in upper part; prosternum bare; 1 long postpronotal and 2–3 shorter setae; about 14–24 biserial acrostichals; 8–10 uniserial dorsocentrals (acrostichals and dorsocentrals about 0.15 mm long in middle of row), ending in 1–2 prescutellars; 1 presutural intra-alar; 1 presutural supra-alar (usually 1 seta between presutural intra-alar and presutural supra-alar); 3 notopleurals (0–2 rather long setae on anterior part of notopleuron); 1 postsutural supra-alar (with 1–2 additional setae); 1 postalar; 2 long and 2 shorter scutellars; laterotergite with several black setae. **Legs** including coxae yellow, microtrichose, black to brown setose. Fore femur with very fine anteroventral setae (nearly half as long as femur width), posteroventral setae half as long as anteroventrals. Fore tibia with almost uniform setation dorsally (shorter than tibia width), setae not differentiated except preapicals, ventral setae short. Mid femur with rows of anteroventral and posteroventral setae (anteroventrals nearly as long as femur width, posteroventrals somewhat longer). Mid tibia with 1–2 scarcely differentiated, short, anterodorsal setae in basal half (nearly as long as tibia width), other setae short; ventrally with two rows of short spine-like setae. Hind femur with anteroventral setae slightly shorter than femur width and with somewhat longer posterior setae over entire length of femur. Hind tibia with several posterodorsal setae nearly as long as tibia width, anterodorsals less differentiated; ventral setae short but spine-like in middle of anteroventral row; 1 short seta in posteroapical comb. Basal tarsomeres of all legs thin, short setose, fore basitarsus elongated. **Wing** clear, stigma scarcely distinct; veins yellowish, CuA+CuP almost complete or weakened in middle. Costal seta short or absent. Axillary excision slightly acute. Measurements: M_2_/dm = 1.3–1.6, M_4_ ap/mp = 1.8–1.9, wl/ww = 2.7–2.8. Halter yellow, calypter yellow with brown fringes. **Abdomen** yellowish brown to yellow, rather faintly light grey microtrichose. Chaetotaxy: setae all brown; posteromarginal setae on sides of tergites 2–4 subequally long as corresponding segment, on segments 5–6 short; discal setae shorter than marginals; dorsum of tergites shorter setose. **Terminalia** as in Figure 3B (not macerated), entirely brownish yellow to yellow. Cercus elongated, broad, nearly parallel-sided (lateral view), with small subbasal projection ventrally; bearing only minute setulae subapically. “Subcercal” process long and bifurcated at apex; with short fine setae apically. Epandrial lamella subtriangular, narrow, ending in yellow, shiny, hook with black spine at tip (lateral view); bearing rather long, fine, brownish setae. Hypandrium subtriangular apically (ventral view). Phallus thickened at base, remaining portion slender, bent before apex.

**Female.** Length: body 5.6 mm, wing 6.0 mm. Similar to male but with the following exceptions. Eyes broadly dichoptic, dorsal ommatidia smaller than ventral ommatidia. Ocellar setae about as long as frons. Frons slightly yellowish translucent just above antennae, 0.20 mm broad and 0.18 mm long, with 2–3 setae on each side. Face 0.18 mm broad and 0.20 mm long. Labrum slightly longer than head height. Thorax as in male, only setae slightly shorter (acrostichals and dorsocentrals about 0.10 mm long). Legs similarly coloured as in male but shorter setose. Fore femur short setose, ventral setae at most 1/3 as long as femur width. Fore tibia short setose. Mid femur with anteroventral and posteroventral setation nearly half as long as femur width. Mid tibia with less differentiated setae than in male, ventrally short and fine setose. Hind femur with setae nearly 2/3 as long as femur is high. Hind tibia thin, with several posterodorsal setae shorter than tibia width, anterodorsals less differentiated (except preapicals), ventral setae short. Basal tarsomere of all legs slender, short setose. Wing dark brown and broadened, stigma equally dark. Measurements: M_2_/dm = 1.7, M_4_ ap/mp = 3.2, wl/ww = 2.0. Abdomen yellow to yellowish brown, entirely faintly grey microtrichose. Hind marginal setae on tergites 2–3 1/3 as long as their corresponding segment, on remaining segments they shorter, discal setae shorter than marginals.

**Differential diagnosis:** *Rhamphomyia* (*P.*) *amurensis* sp. nov. is a member of the *R.* (*P.*) *tipularia* group, which is especially species rich in the Northeast Palaearctic (and also in North America), where many species still remain undescribed. Species of this group have legs usually at least partly yellow, uniserial (sometimes almost biserial) dorsocentral setae, costal seta short to absent and reduced prothoracic setae, and females have often dark brown or even broadened wing and conspicuously pale (yellowish brown, yellow to white) abdomen. We could distinguish two species complexes in this group: *R. tipularia* complex and *R. hilariformis* complex differing in male genitalia (males of the former have elongated cercus which is longer than epandrium). The male of the new species described above can be readily distinguished according to the characteristic shape of male genitalia. However, the female (taken without male) can be scarcely identified with certainly because females of several other still undescribed species are very similar. However, the species described above differs from known species of the *R*. *tipularia* group in having yellow basal antennomeres, uniserial dorsocentrals, brown fringes on calypter and thin and sparsely setose mid and hind femora (*R. epandriata* sp. nov. female has pale fringes on calypter and *R. globulicauda* sp. nov. has dense and long posteroventral setation on mid and hind femora; females of several undescribed species have either biserial dorsocentrals or dark basal antennomeres). A very similar species occurs also in Alaska; however, it differs in details of the male genitalia.

**Distribution:** Russia (Amurskaya Province, Yakutia).

**Dates of occurrence:** June.

### 3.4. Rhamphomyia (Pararhamphomyia) angustitibia sp. nov.

Zoobank link: urn:lsid:zoobank.org:act: 41380E4E-8273-4DCF-ACF4-D4195C5F16FA

(Figure 4A,B)

**Type material: HOLOTYPE** ♂, labelled: [in Cyrillic; **RUSSIA**, Sakha (Yakutia)] r. [=river] Indigirka/lower flow of Ystyn-Yuryakh River/Momskiy District [66°27′ N 143°13′ E]/1.vii.1976/V. Kovalev (ZMMU).

**Figure 4:**
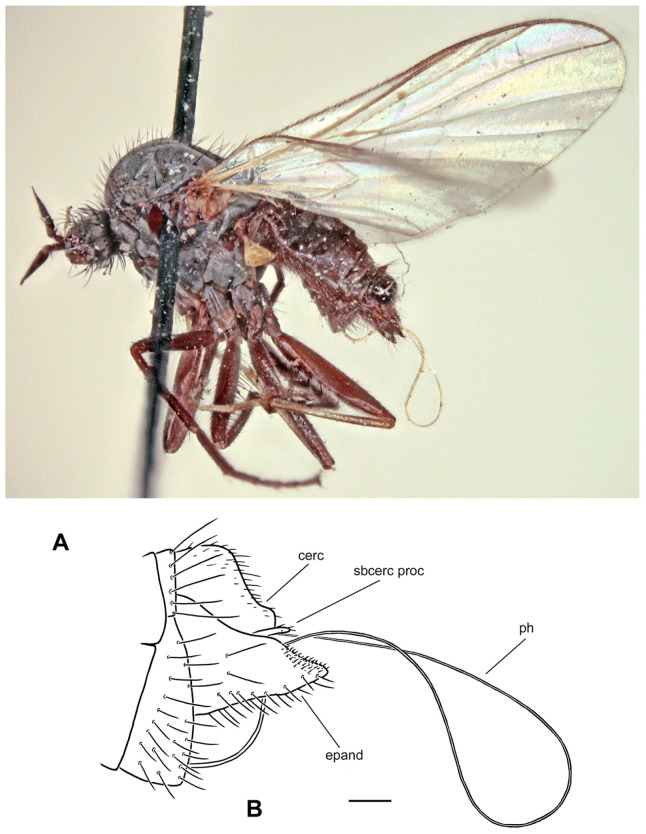
*Rhamphomyia* (*Pararhamphomyia*) *angustitibia* sp. nov., male holotype. (**A**) = Habitus, lateral view. (**B**) = Terminalia, lateral view. Abbreviations: cerc—cercus, epand—epandrial lamella, ph—phallus, sbcerc proc—subcercal process. Scale bar 0.1 mm.

**Diagnosis:** Small species of *Rhamphomyia* (*Pararhamphomyia*) with broadly dichoptic eyes of male, very slender and short pilose mid and hind tibiae.

**Etymology:** The species is named after peculiarly slender mid and hind tibiae.

**Description: Male** (Figure 4A). Length: body 2.5 mm (without genitalia), wing 3.3 mm. **Head** regions black, light grey microtrichose, black setose. Eyes broadly dichoptic, dorsal ommatidia smaller than ventral ommatidia. Frons 0.13 mm broad and 0.18 mm long, with 1 seta on each side. Ocellar setae slightly longer than frons, ocellar triangle with 2 additional setae on posterior margin. Face subequally broad as frons and 0.20 mm long, bare. Occiput rather sparsely covered with fairly strong setae; postocular row incomplete, absent in ventral part. Antenna with scape and pedicel brown, postpedicel and stylus black; scape and pedicel short setose (longest setae 0.10 mm long); antennomere ratio = 10:8:27:9. Labrum brown, shiny, as long as head height. Palpus brown, bearing a few setae. Gena narrow and microtrichose. **Thorax** brownish black, almost light grey microtrichose, without vittae or with indefinite darker vittae down rows of setae viewed from behind. All setae black. Chaetotaxy: proepisternum with 1–2 long setae on lower part, bare on upper part; prosternum bare; postpronotal lobe with 1 long and about 15 shorter strong setae of subequal lengths; 22 biserial acrostichals; 13–15 irregularly biserial (almost uniserial in postsutural part of mesoscutum) dorsocentrals, ending in 3 prescutellars; acrostichals and dorsocentrals moderately strong, about 0.15 mm long in middle of rows and several setae present also in prescutellar depression; about 10–15 setae outside of dorsocentrals in presutural part of mesoscutum, presutural intra-alar seta not differentiated, presutural supra-alar is only slightly stronger; 3 notopleurals (and 5 finer short setae on anterior part of notopleuron); 1 postsutural supra-alar (with 2 setae on prealar area); 1 long and 1 short postalar; 4 scutellars (lateral pair slightly shorter than apical); laterotergite with numerous setae. **Legs**, including coxae, mostly brown, microtrichose; mid femur with shiny anterior stripe in basal half, hind femur with similar stripe over entire length, hind tibia and tarsus whitish yellow. Dark parts of legs covered with brown setae and pale parts with yellow setae. Fore leg short setose. Mid femur almost bare anteroventrally; posteroventrally with irregular row of setae in middle part of femur (nearly 1.5× as long as femur width); dorsal setae short. Mid tibia slender (diameter about 0.05 mm in middle), with short dorsal setae and dense short ventral pubescence. Hind femur slightly swollen in middle part; basal ventral 2/3 covered with irregularly arranged setae, 3 longest setae nearly as long as femur width, remaining setae rather spinose and shorter; dorsal setae short, posteroventral surface bare; apical third of femur only pale tomentose ventrally, without setae. Hind tibia conspicuously thin (about 0.04 mm in middle), with very short pale setae dorsally and with fine pubescence ventrally; 1 moderately long seta in posteroapical comb. Basitarsus of all legs slender, short setose, mid basitarsus with short ventral spines. **Wing** clear, stigma hyaline, veins pale; vein CuA+CuP absent in apical part. Basal costal seta present. Axillary excision slightly obtuse. Measurements: M_2_/dm = 1.3–1.4, M_4_ ap/mp = 1.6–1.7, wl/ww = 2.6. Halter yellow with brownish stem; calypter brownish yellow with dark fringes. **Abdomen** brown, brownish grey microtrichose, covered with dark setae. Chaetotaxy: posteromarginal setae on sides of tergites half as long as their corresponding segment, discal setae subequal to marginals; dorsum of tergites with setae almost as long as setae on sides. **Terminalia** as in Figure 4B (not macerated); cerci and epandrium concolorous with abdominal tergites, black setose, cercus partly shiny. Epandrial lamella triangular (lateral view). Hypandrium invisible in situ. Cercus broadly rounded at apex. “Subcercal” process narrow. Phallus very long and thin.

**Female.** Unknown.

**Differential diagnosis:** *Rhamphomyia* (*P.*) *angustitibia* sp. nov. is very similar to *R. praestans* Frey in having broadly dichoptic eyes, very slender and short pilose mid and hind tibiae as well as setose presutural depression. However, *R. praestans* is a larger species (wing over 4.0 mm) with abdomen shiny at least on last tergites and the postpedicel is about 4 times longer than the style. There are also slight differences in terminalia in both species: in *R. praestans* the cercus is more narrowed apically and “subcercal” process is broader. The female remains unknown, but we believe that it should be recognised from other *Pararhamphomyia* according to setose presutural depression and smaller size than *R. praestans*.

**Distribution:** Russia (Yakutia).

**Dates of occurrence:** July.

### 3.5. Rhamphomyia (Pararhamphomyia) basitarsata sp. nov.

Zoobank link: urn:lsid:zoobank.org:act: 3906B5D9-EA60-464C-9CAA-D9CC82517B2B

(Figure 5A,B)

**Type material: HOLOTYPE** ♂, labelled: RUS [Russia], Primorsky reg, 42.7 N/131.1 E, Andreevka env,/1–3.viii, 2018, N. Vikhrev (ZMMU). **PARATYPES: CHINA. Jilin Prov.:** Erdaocun, 800 m, 42.421 N, 128.077 E, 25–26.vi.2017, E. Jendek and O. Šauša leg. (3 ♂, 3 ♀, CULSP); Jiaohe env. 95 km NNE of Jilin, 43.95 N, 127.7 E, 400–600 m, 27.vi.–5.vii.2017, E. Jendek and O. Šauša leg. (1 ♀, CULSP). **RUSSIA. Amurskaya Prov.:** Samodon, 100 km W Svobodny, mixed forest, 6.viii.1959, Zinovjev (2 ♂, 4 ♀, ZISP). **Primorskiy Terr.:** Kamenushka, 10.vii.1984, A. Shatalkin (1 ♂, ZMMU); Kedrovaya Pad’, 2.vii.1984, A. Shatalkin (1 ♂, ZMMU); Andreevka env., 42.7 N 131.1 E 26–31.vii.2018, N. Vikhrev (1 ♂, 1 ♀, ZISP); same locality as holotype, 1–3.viii, 2018, N. Vikhrev (1 ♂, ZISP); same locality as holotype, 25–30.vi.2014, N. Vikhrev (1 ♂, ZISP); Anisimovka, 450 m, 43.13 N 132.8 E, 21–24.vii.2018, N. Vikhrev (1 ♂, ZISP).

**Diagnosis:** Medium-sized (body about 4.2–5.5 mm) black and black setose species of *Rhamphomyia* incertae sedis with setose prosternum and dark halter. Male: hind femur ventrally without setae. Female: frons shiny.

**Etymology:** The species is named after the peculiarly long hind basitarsus.

**Figure 5:**
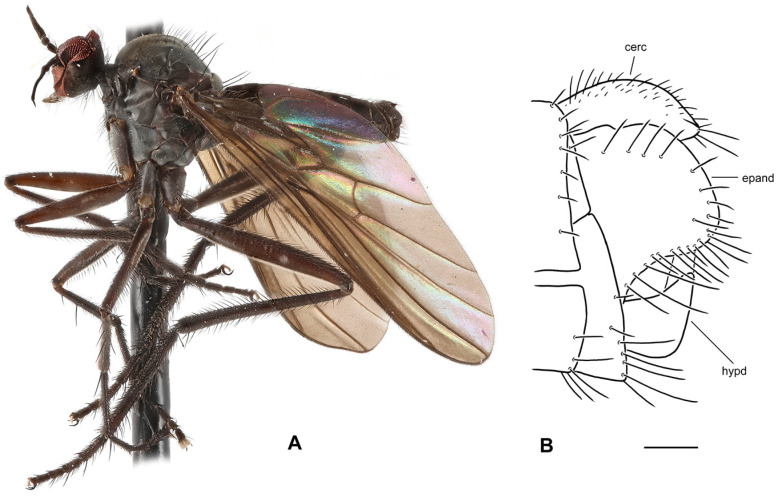
*Rhamphomyia* (*Pararhamphomyia*) *basitarsata* sp. nov., male holotype (**A**) = Habitus, lateral view. (**B**) = Terminalia, lateral view. Abbreviations: cerc—cercus, epand—epandrial lamella, hypd—hypandrium. Scale bar 0.1 mm.

**Description: Male** (Figure 5A). Length: body 4.2–5.5 mm, wing 4.2–4.7 mm. Head regions blackish brown, grey microtrichose (except noted), black setose. Eyes holoptic, upper ommatidia enlarged. Frons bare. Ocellar setae fine, half as long as frons. Face parallel-sided, about 0.13 mm broad and 0.25 mm long, bare. Occiput brown microtrichose, rather dense and irregular setose; occipital setae short behind eyes and long towards neck, postocular row incomplete, almost not differentiated. Antenna with scape and pedicel brown, rather long setose (longest setae about 0.17 mm long), postpedicel and stylus back; antennomere ratio = 12:9:38:8. Labrum brown, shiny, 2/3 as long as head is high. Palpus brown, short, with several short setae. Gena narrow and microtrichose, clypeus microtrichose. **Thorax** blackish brown, brown microtrichose; mesoscutum in dorsal view almost uniformly blackish brown, in posterior view with somewhat darker vittae down rows of acrostichal and dorsocentral setae. All setae black. Chaetotaxy: proepisternum with about 8 setae on lower part, bare in upper part; prosternum with row of 4–5 setae on anterior part; 1 strong postpronotal (with additional 4–5 much shorter, fine setae); about 16 biserial, fine acrostichals; about 15 biserial (almost uniserial on anterior part) dorsocentrals subequally long as or slightly longer than acrostichals (0.15 mm in middle of rows), ending in 1 strong prescutellar seta; 1–2 fine presutural intra-alar(s); 1–2 presutural supra-alar(s) (with 2–3 additional short, fine setae nearby); 2 notopleurals (2 additional fine setae on anterior part of notopleuron); 1 postsutural supra-alar (0–1 seta on prealar area), 1 long and 1 small postalar, 4 scutellars (outer pair half as long as inner pair); laterotergite with numerous setae. **Legs**, including coxae, brown, microtrichose, black setose. Fore femur with short setae dorsally; bearing minute, fine, scarcely distinct setae anteroventrally and posteroventrally. Fore tibia with short and fine uniform posterodorsal setation about as long as or shorter than tibia width (contrastingly strong but short preapicals), faintly pubescent ventrally. Mid femur with strong, spinose, anteroventral setae scarcely 1/3 as long as femur width (with a few fine but longer anteroventrals about base) and with similarly strong, spinose, longer posteroventrals (nearly as long as femur width); dorsal setae short. Mid tibia with 2–3 anterodorsal and posterodorsal long setae (2–3× as long as tibia width) and with 1 unusually strong, dorsal, preapical seta, which is about 0.40 mm long (almost 4× as long as tibia width) and almost 0.02 mm thick; bearing anteroventral row of short, spinose setae (1/3 as long as tibia width, similar to anteroventral on mid femur but shorter) and posteroventral row consisting of rather strong, equally short setae. Hind femur short setose dorsally; only pale tomentose ventrally; with moderately long, fine setae in basal half posteriorly. Hind tibia with short setae dorsally (nearly as long as tibia width); bearing row of anteroventral, short, spine-like setae (similar to those on mid tibia) and row of posteroventral, much longer, fine setae (longest setae slightly longer than tibia width); 1 long seta in posteroapical comb. Fore basitarsus slender, only faintly pubescent ventrally, with slightly elongated setae dorsally; mid basitarsus slender, with short ventral spines, short setose dorsally; hind basitarsus very long (almost 1.5 mm long, much longer than remaining tarsomeres combined), slender, with short ventral spines, covered with moderately long setae dorsally; tarsomeres 1–4 of all legs with circlet of rather long subapical setae. **Wing** membrane brown, stigma only slightly darker than surrounding membrane, veins dark brown; vein CuA+CuP complete, slightly S-shaped, only indistinctly weakened or depigmented about middle. Costal seta present, axillary excision acute (60°). Measurements: M_2_/dm = 1.7, M_4_ ap/mp = 2.8–2.9, wl/ww = 2.7. Halter brown, calypter dark brown with black fringes. **Abdomen** brown, microtrichose, sides of basal tergites and sternites rather subshiny. Chaetotaxy: setae all dark; hind marginal setae on sides of tergite 2 almost as long as segment, on tergite 3 half as long as segment, on remaining segments they are scarcely 1/3 as long as their corresponding segment, discal setae slightly shorter than marginals; dorsum of tergites with shorter setae. **Terminalia** (Figure 5B, not macerated) simple, concolorous with abdominal tergites, black setose. Epandrial lamella rather subtriangular (lateral view), with short, fine setae apically. Hypandrium rather large, shiny and exposed. Cercus with lateral preapical protuberance bearing several long setae. “Subcercal” process obviously absent. Phallus thick and short, hidden between epandrial lamellae.

**Female.** Length: body 4.9–5.4 mm, wing 4.0–4.7 mm. Dichoptic, ommatidia of subequal size. Frons about 0.17 mm wide, shiny except ventral part. Thorax as in male (colour and chaetotaxy), only setae distinctly shorter. Legs differently setose. Fore femur with dense short ciliation dorsally (almost subpennate). Fore tibia short setose. Mid femur with subpennate to slightly pennate setae anterodorsally and posteroventrally. Mid tibia with several short setae anterodorsally and posterodorsally. Hind femur similarly setose as mid femur. Hind tibia short setose, dorsal setae poorly differentiated and subequally long as tibia width. Basitarsus of all legs thin and short setose, hind basitarsus elongated as in male. Wing as in male. Abdomen brown, entirely microtrichose, with short setae (posteromarginal lateral setae on tergites 2–4 scarcely one-third as long as corresponding segment).

**Differential diagnosis:** *Rhamphomyia* (*P.*) *basitarsata* sp. nov. is quite an isolated species among the members of the genus *Rhamphomyia*. Several characters (e.g., complete vein CuA+CuP and acute axillary excision) resemble subgenus *Rhamphomyia* s. str.; however, the proepisternal depression is bare. The new species can be easily recognised from all other Palaearctic *Rhamphomyia* by setose prosternum, entirely dark setose body and dark halter (similar combination occurs only in *R*. *pseudocrinita* Strobl, however, this species has setose proepisternal depression). The only other Palaearctic species sharing these characters is *R. trichopleura* Saigusa [25]. However, this species is in all other characters dissimilar, e.g., it has uniserial dorsocentral setae and setose proepimeron.

**Distribution:** China (Jilin Province), Russia (Amurskaya Prov., Primorskiy Ter.).

**Dates of occurrence:** July–August.

### 3.6. Rhamphomyia (Pararhamphomyia) bifurcata sp. nov.

Zoobank link: urn:lsid:zoobank.org:act: A1BC2258-9A9E-46C3-8623-32015447EAB0

(Figure 6A,B)

**Type material: HOLOTYPE** ♂, labelled: [in Cyrillic, Russia] Amurskaya obl. [=oblast, province]/g. [=gorod, town] Zeja [53°44′ N 127°15′ E]/3.vii.1981/A. Ozerov (ZMMU). **PARATYPES: RUSSIA. Amurskaya Prov.:** same locality as holotype, 1.vii.1981, A. Shatalkin (1 ♂, ZMMU). **Buryatia:** Bargusinskiy Nature Reserve, 15.vii.1965, Negrobov (1 ♂, 1 ♀, ZISP). **Khabarovskiy Terr.:** promysel Ozernakh, estuary of Amur, 20–26.vi.1915, Chernavin (1 ♂, ZISP). **Nenets AO:** Naryan-Mar, 4.viii.1978, Gorodkov (1 ♂, ZISP). **Yakutia:** Indigirka River, env. Ust-Nery, 13.vi.1976, V. Kovalev (1 ♂, 1 ♀, CULSP and ZMMU); 232 km of Khandyga, 1650 m, 27.vi.1985, A. Barkalov (1 ♂, ZISP).

**Diagnosis:** Medium-sized species (body about 4.5 mm) of *Rhamphomyia* (*Pararhamphomyia*) with black setose body, brown microtrichose mesoscutum, 1–2-serial dorsocentral setae, uniformly dark legs, brownish wing and halteres at least with brownish stem. Male: epandrium bifurcated at apex, both lobes bearing spines near tip. Female: mid and hind femora with flattened setae.

**Etymology:** The species is named after the bifurcated tip of male epandrium.

**Figure 6:**
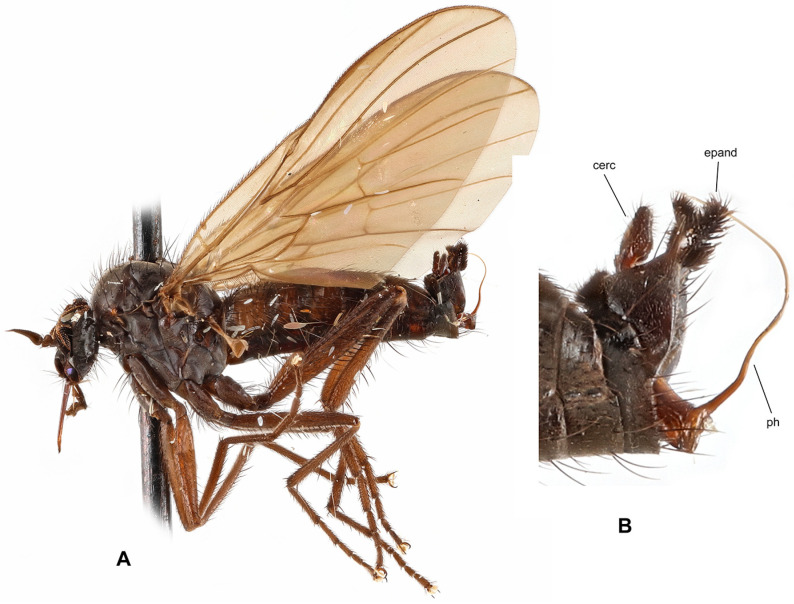
*Rhamphomyia* (*Pararhamphomyia*) *bifurcata* sp. nov., male. (**A**) = Habitus, lateral view. (**B**) = Terminalia, lateral view. Abbreviations: cerc—cercus, epand—epandrial lamella, ph—phallus.

**Description: Male** (Figure 6A). Length: body 4.5 mm (without genitalia), wing 4.5–5.0 mm. **Head** regions black to brownish black, grey microtrichose black setose. Eyes holoptic, upper ommatidia much larger. Frons bare. Ocellar setae fine, 1/3 as long as frons; ocellar triangle with 2 pairs of additional setae in posterior part. Face about 0.19 mm broad ventrally and 0.13 mm long, bare; clypeus microtrichose (except dorsalmost part). Occiput rather densely covered with setae; postocular row complete but distant from eye margin in middle third. Antenna black (scape and pedicel very slightly paler), scape and pedicel setose (longest setae about 0.09 mm long); antennomere ratio = 8:9:33:10. Labrum brown, shiny, as long as or slightly shorter than head height. Palpus brown, short, bearing a few setae (longest setae about 0.20 mm long). Gena narrow, microtrichose. **Thorax** brownish black, mesoscutum brown microtrichose, in dorsal view without vittae or in posterior view with scarcely distinct darker vittae under rows of acrostichal and dorsocentral setae. All setae black. Chaetotaxy: proepisternum with 6–10 setae on lower part and 1–2 setae in upper part; prosternum bare; postpronotal lobe with 1 long and 8–9 shorter setae; 12–18 biserial acrostichals; 10–14 irregularly 1–2-serial dorsocentrals ending in 2–3 long prescutellars (acrostichals and dorsocentrals about 0.20–0.25 mm long in middle of rows); 1 presutural intra-alar (with 3–5 additional setae in presutural part outside of dorsocentrals); 2–4 notopleurals (2–4 rather strong additional setae on anterior part of notopleuron); 2 postsutural supra-alars and 2–4 setae in prealar area; 1 postalar; 4 scutellars (lateral pair half as long as apical pair, sometimes 1–2 additional setae present); laterotergite with numerous setae. **Legs**, including coxae, brown, microtrichose, black setose. Fore femur with rows of short anteroventral and posteroventral setae (half as long as femur width); dorsal setation short. Fore tibia with well-differentiated 1–2 anterodorsal and 3–4 posterodorsal setae (1.5× as long as tibia width); fine ventral setae short. Mid femur with spinose anteroventral setae (about half as long as femur width) and somewhat longer, equally spinose posteroventrals (nearly as long as femur width); dorsal setation short. Mid tibia with 1–2 anterodorsal and 2–3 posterodorsal long setae nearly twice as long as tibia width (small ciliation about as long as tibia width), ventrally with short and fine setation (without rows of setae). Hind femur with irregular rows of anteroventral and posteroventral spinose setae shorter than femur width (longest setae about 2/3 as long as femur width), those in anteroventral row somewhat clustered some distance before tip; dorsal setation short. Hind tibia slightly swollen in apical half, with several irregularly arranged anterodorsal and posterodorsal setae slightly longer than tibia width; ventral setation short and fine, similarly as in mid tibia; 1 relatively long seta in posteroapical comb. Basitarsus of all legs slender and short setose, fore and hind basitarsi with short ventral spines. **Wing** light brownish, stigma brown, veins yellowish brown; vein CuA+CuP complete but weakened in middle. Basal costal seta present. Axillary excision acute (60°). Measurements: M_2_/dm = 1.3–1.4, M_4_ ap/mp = 1.6–1.8, wl/ww = 2.5–2.6. Halter with yellow knob and brownish stem or fully brown; calypter brownish yellow with dark fringes. **Abdomen** brown, microtrichose, all setae dark. Chaetotaxy: posteromarginal setae on sides of tergites nearly as long as their corresponding segment; discal setae (except segment 2) much shorter; dorsum of tergites short setose. **Terminalia** as in Figure 6B; concolourous with abdominal tergites, black setose. Epandrial lamella bifurcated at apex (lateral view), both lobes bearing spines near tip, those on ventral lobe very strong; epandrium and “subcercal” process with inside going projections. Cercus deeply concave dorsally, with scattered setulae. Phallus well exposed, mostly this and gently curved, thickened at base.

**Female.** Length: body 4–4.2 mm, wing 4.3–4.5 mm. Similar to male, except as follows. Eyes broadly dichoptic, upper ommatidia slightly smaller than ventral ommatidia. Frons 0.19 mm broad and 0.20 mm long, with a few fine setae on each side. Face equally broad as frons but only 0.14 mm long. Antennomere ratio = 9:9:36:9. Ocellar setae strong and as long as frons. Occiput short and sparse setose. Labrum slightly longer than head height. Thorax coloured and setose as in male, only setae shorter (both acrostichals and dorsocentrals about 0.15 mm long). Legs including coxae similarly coloured as in male, however, differently setose. Fore femur as in male. Fore tibia with several short but distinct anterodorsal and posterodorsal setae (subequally long as tibia width). Mid femur with irregular rows of anteroventral and posteroventral setae slightly shorter than femur width and rather flattened, dorsal setation short and very slightly flattened. Mid tibia with 3–4 pairs of setae dorsally nearly twice as long as tibia width, ventral setae short as in male but finer. Hind femur with irregular dense row of anteroventral setae nearly as long as femur width, posteroventral setae slightly shorter but slightly flattened, dorsal setation short. Hind tibia with 4–5 anterodorsal and 5–6 posterodorsal setae slightly longer than tibia width. Basal tarsomeres of all legs slender, short setose, with short ventral spines. Wing darker brown than in male, especially dark along basal veins and along costal margin, stigma equally dark. Measurements: M_2_/dm = 1.4, M_4_ ap/mp = 1.9–2.0, wl/ww = 2.4. Halter, CuA+CuP and axillary excision as in male, calypter brown. Abdomen brown, microtrichose (tergites 2–4 rather thinly), posteromarginal setae on sides of tergites 2–4 nearly as long as their corresponding segment, on tergite 5 these setae are 2/3 as long as segment, on remaining tergites, shorter and finer; discal setae much shorter than marginals.

**Differential diagnosis:** *Rhamphomyia* (*P.*) *bifurcata* sp. nov. belongs to species-rich group of *Pararhamphomyia* with 1–2-serial dorsocentral setae, dark legs and entirely black setose body. The most allied species is probably *R. fuscipennis* (Zetterstedt), which differs from the above-described species in having 6 scutellar setae and the male has longer and differently shaped phallus. The female can be recognised by slightly flattened setae on mid and hind femora, yellow halters, microtrichose abdomen, setose proepisternal depression and acute axillary angle. If the flattened setae is overlooked, it resembles *R. fuscipennis*. However, this species has paler calypter, shorter setose abdomen and hind tibia with distinct anteroventral setae. Another allied species is *R. angulifera* Frey. It should be noted that a very similar, and maybe identical, species occurs in the Nearctic region (Alaska).

**Distribution:** Eurasia: Russia (Amurskaya Prov., Buryatia, Khabarovskiy Terr., Nenets AO, Yakutia). North America: ?Alaska.

**Dates of occurrence:** June–July.

### 3.7. Rhamphomyia (Pararhamphomyia) epandriata sp. nov.

Zoobank link: urn:lsid:zoobank.org:act: AA54A448-7933-4106-B314-F12697B945A7

(Figure 7A,B)

**Type material: HOLOTYPE** ♂, labelled: [in Cyrillic; **RUSSIA**, Yakutia-Sakha], r. [=reka, river] Indigirka/ust. r. [=ust’e reki, mouth of river] Injali [65°14′ N 143°08′ E]/24.vi.1976/V. Kovalev (ZMMU). **PARATYPES: RUSSIA. Yakutia:** same locality as holotype, 23.vi.1976, V. Kovalev (1 ♀, CULSP); Ust-Kuyga, Yana, 27.vii.1974, Gorodkov (1 ♂, ZISP).

**Diagnosis:** Medium-sized (body 4–4.5 mm) species of *Rhamphomyia* (*Pararhamphomyia*) *tipularia* group; antenna with scape and pedicel brownish, dorsocentral setae 1–2-serial, calypter pale fringed. Male: basitarsus of fore legs slightly thickened but not elongated, mid tibia with long setae; cercus enlarged, with finger like projection ventrally on apex. Female: legs short setose.

**Etymology:** The species is named to stress enlarged cercus.

**Figure 7:**
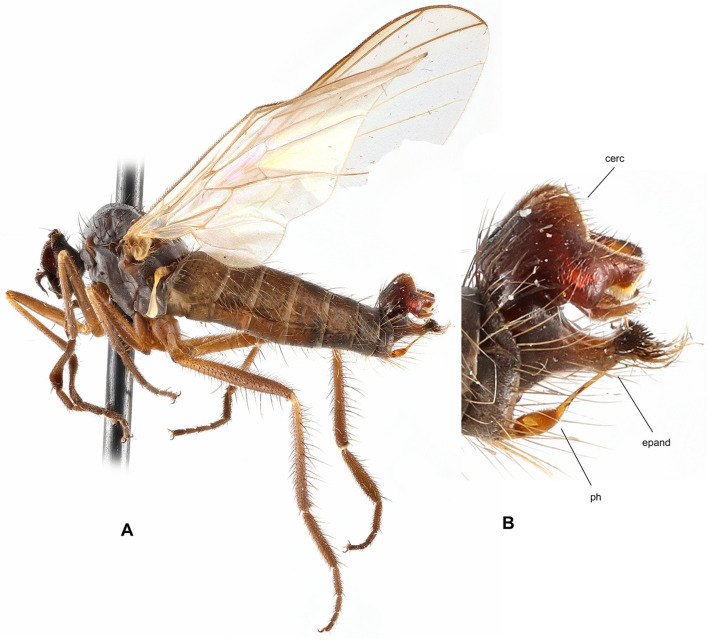
*Rhamphomyia* (*Pararhamphomyia*) *epandriata* sp. nov., male. (**A**) = Habitus, lateral view. (**B**) = Terminalia, lateral view. Abbreviations: cerc—cercus, epand—epandrium, ph—phallus.

**Description: Male** (Figure 7A). Length: body 4.1 mm (without genitalia), wing 5.4 mm. **Head** regions black to brownish black, light grey microtrichose. Eyes holoptic, upper ommatidia much larger than lower ommatidia. Frons bare. Ocellar setae black, scarcely half as long as frons; ocellar triangle with additional short setae only in posterior part. Face about 0.15 mm broad ventrally and 0.20 mm long, bare; clypeus microtrichose. Occiput sparsely black setose, occipital setae almost confined to 2 irregular rows. Antenna with brownish scape and pedicel, postpedicel and stylus blackish brown, scape and pedicel short setose; antennomere ratio = 12:9:40:10. Labrum brown, shiny, about as long as head height. Palpus brown, short, bearing a few setae. Gena narrow, microtrichose. **Thorax** brownish black, light grey microtrichose, mesoscutum without vittae. Setae mostly black to brown, only laterotergite with partly pale setae as well as calypter pale fringed. Chaetotaxy: proepisternum with 2 setae on lower part, bare on upper part; prosternum bare; postpronotal lobe with 1 long and 5 shorter setae; about 20 biserial acrostichals; 10 irregularly 1–2-serial dorsocentrals ending in 2–3 prescutellars (acrostichals and dorsocentrals about 0.20 mm long in middle of row); 1 presutural intra-alar; 1 presutural supra-alar (0–1 additional seta between presutural intra-alar and presutural supra-alar); 3 notopleurals, (3 rather long setae on anterior part of notopleuron); 1 supra-alar (2 rather strong setae on prealar area); 1 postalar; 4 scutellars; laterotergite intermixed pale and brown setae. **Legs**, including coxae, brown to yellowish brown, fore trochanters and narrow bases of mid and hind femora yellow. Legs entirely microtrichose and predominantly brown setose. Fore femur bare ventrally and short setose dorsally. Fore tibia with equally short posterodorsal and ventral setation (shorter than tibia width), at most with 1 short submedian anterodorsal seta. Mid femur with rather long and numerous anteroventrals in about basal 2/3, minute setae in apical 1/2; posteroventrals short throughout, slightly shorter than femur width. Mid tibia with 5 long setae dorsally (about 0.30 mm long or 3× as long as tibia width), other setae short and fine except somewhat spinose, short, anteroventral setae occurring in apical half of tibia. Hind femur with anteroventral and posteroventral setae short in basal half of femur, but in apical half they are nearly twice as long as femur width, also anterior and posterior setae in the apical part of the femur relatively long. Hind tibia with several irregularly arranged setae dorsally nearly twice as long as tibia width, anteroventrals and posteroventrals about as long as tibia width, posteroventrals in basal third fine and almost upright standing (contrastingly with those in more distal part of tibia much shorter and adjoined); rather short seta in posteroapical comb. Fore basitarsus slightly swollen, short setose; mid basitarsus slender, short setose; hind basitarsus slender, dorsally with 3 setae nearly twice as long as its diameter. **Wing** clear, stigma scarcely distinct (hyaline), veins yellowish brown; vein CuA+CuP depigmented in basal 2/3 but present in apical 1/3 near wing margin. Costal seta present, axillary excision 90º. Measurements: M_2_/dm = 1.6, M_4_ ap/mp = 2.2–2.3, wl/ww = 2.8. Halter yellow, calypter yellow with pale fringes. **Abdomen** brown (contrastingly lighter than thorax), apical parts of genital lamellae yellowish brown, light grey microtrichose (only cercus is shiny). Chaetotaxy: setae brown but very pale, almost yellow on segment 8 and genital lamellae; hind marginal setae almost as long as their corresponding segment, discal setae subequally long as marginals; dorsum of tergites with long setae. **Terminalia** as in Figure 7B; epandrium yellowish brown, cerci reddish yellow. Epandrial lamella elongate, subtriangular (lateral view), bearing subapical dorsal bump with short black spines and long curved pale setae on apex. Cercus enlarged, with finger-like projection ventrally on apex; bearing minute setulae, finger-like projection pale yellow tomentose at apex (posterior view). “Subcercal” process short, bifurcate. Phallus mostly thin, gently curved, thickened at base.

**Female.** Length: body 6.5 mm, wing: 7.0 mm. Similar to male, except as follows. Eyes broadly dichoptic, dorsalmost ommatidia slightly smaller than ventral ommatidia. Ocellar setae 2/3 as long as frons. Frons 0.22 mm broad and 0.30 mm long, with 3–4 setae on each side (nearly 0.10 mm long). Face as broad as frons and 0.20 mm long. Antennomere ratio = 12:10:43:11. Labrum as in male. Thorax as in male, only setae much shorter (acrostichals and dorsocentrals about 0.10–0.15 mm long) and more numerous (about 20 dorsocentrals and 30 acrostichals, 6 short scutellars), colour of setae as in male. Legs similarly coloured as in male (even trochanters and base of femora yellow as in male) but different setose. Fore femur short setose, but anteroventrals and posteroventrals present. Fore tibia very short setose, setae scarcely 1/3 as long as tibia width. Mid femur very short setose, anteroventrals and posteroventrals scarcely 1/4 as long as femur width, only several ventral setae on base of femur slightly longer. Mid tibia short setose, several setae (mostly in anteroventral and anterodorsal position) half as long as tibia width. Hind femur short setose, anteroventrals much shorter than femur is wide, posteroventrals almost absent. Hind tibia thin, several dorsal setae and 1–2 anteroventral setae shorter than tibia width, ventral setae short. Basitarsus of all legs slender, short setose, fore basitarsus with several long, fine and upright standing ventral setae, mid and hind basitarsi with short ventral spines. Wing pale in basal third and light brown in apical part, stigma concolourous with surrounding membrane, veins and axillary angle as in male, costal seta indistinct. Wing slightly broadened in apical third (broadest place around tip of vein M_4_); M_2_/dm = 1.5, M_4_ ap/mp = 2.0–2.1, wl/ww = 2.5. Calypter and halter as in male. Abdomen brown, entirely grey microtrichose. Hind marginal setae on tergites 2–4 about 1/3 as long as their corresponding segment, on remaining segments they are shorter, discal setae shorter than marginals.

**Differential diagnosis:** *Rhamphomyia* (*P.*) *epandriata* sp. nov. is a member of the *R. hilariformis* complex of the *R.* (*P.*) *tipularia* group (see also discussion under *R. amurensis* sp. nov.). Both sexes of the new species can be primarily distinguished from all known species of this complex in pale fringed calypter. Moreover, the male can be easily recognised in the shape of terminalia, fore basitarsus, which is slightly swollen but not elongated, and in long setose mid tibia as well as the female in short setose legs.

**Distribution:** Russia (Yakutia).

**Dates of occurrence:** June–July.

### 3.8. Rhamphomyia (Pararhamphomyia) globulicauda sp. nov.

Zoobank link: urn:lsid:zoobank.org:act: 89E914B1-EC3A-4296-92A9-460E0B1EA033

(Figure 8A–D)

**Type material: HOLOTYPE ♂**, labelled: [in Cyrillic; **RUSSIA**, Yakutia-Sakha] r. [=reka, river] Indigirka/nizovya r. [lower flow of river] Pystan-Yuryakh, Momskij r-n [=rayon, district; 66°27′ N 143°13′ E]/27.vi.1976/V. Kovalev//somknutye ivy i topolya na beregu [=closed willows and poplars on the shore] (ZMMU). **PARATYPES: RUSSIA. Yakutia:** same data as holotype (2 ♀, ZMMU); Indigirka River, mouth of river Injali, 18.vi.1976, Kovalev (1 ♀, ZMMU); same locality, 24.vi.1976, Kovalev (1 ♂, CULSP); Khaptagatay, 30 km SSE Yakutsk, 30.vi.1974, Nartshuk (1 ♂, ZISP). **Chukotka AO:** Bilibino, 4.vii.1971, Gorodkov (1 ♂, ZISP). **Magadanskaya Prov.:** Ust-Omchug, 30.vi.1971, Gorodkov (1 ♂, 1 ♀, ZISP).

**Figure 8:**
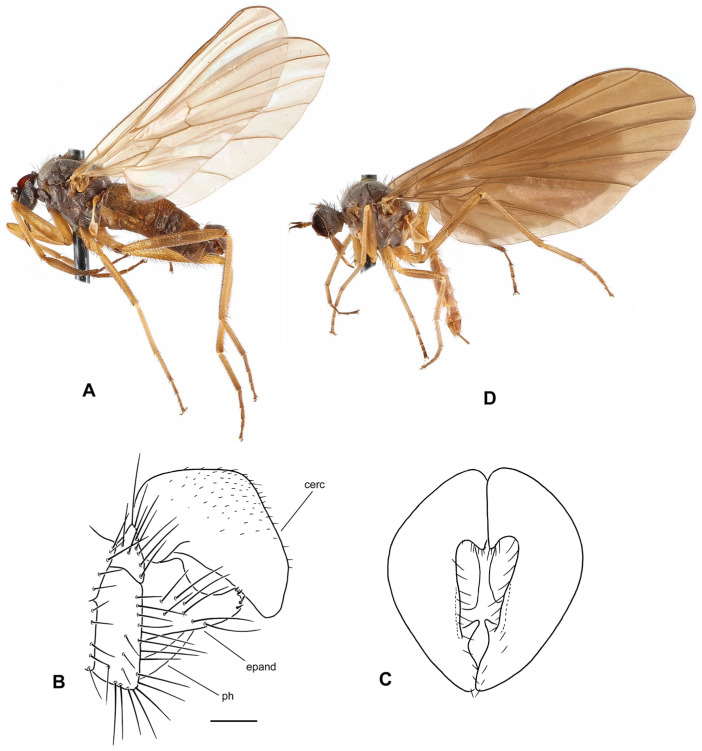
*Rhamphomyia* (*Pararhamphomyia*) *globulicauda* sp. nov. (**A**) = Male habitus, lateral view. (**B**) = Male terminalia, lateral view. (**C**) = Male cerci, dorsal view. (**D**) = Female habitus, lateral view. Abbreviations: cerc—cercus, epand—epandrium, ph—phallus. Scale bar 0.1 mm.

**Diagnosis:** Relatively large (body 5.5–6 mm) species of *Rhamphomyia* (*Pararhamphomyia*) *tipularia* group; antenna with scape and pedicel brownish yellow. Male: basitarsus of fore legs slightly thickened, nearly as broad as fore tibia at apex; cercus semiglobular, much larger than epandrium, with small finger like projection dorsally about base. Female: mid and hind femora with relatively dense posteroventral setation.

**Etymology:** The name of the species is derived from globular cercus.

**Male** (Figure 8A). Length: body 5.5–6.1 mm (without genitalia), wing 7.0–7.8 mm. **Head** regions black to brownish black, light grey microtrichose. Eyes holoptic, upper ommatidia much larger than lower ommatidia. Frons bare. Ocellar setae black, scarcely half as long as frons, ocellar triangle with 1 pair of additional short setae in posterior part. Face about 0.19 mm broad ventrally and 0.22 mm long, bare. Occiput sparsely setose with black and rather strong setae, postocular row incomplete. Antenna with scape and pedicel brownish yellow, short setose, postpedicel and stylus black; antennomere ratio = 12:10:42:14. Labrum brown, shiny, about as long as head height. Palpus brown, short, bearing a few setae. Gena narrow, microtrichose; clypeus microtrichose. **Thorax** brownish black, light brownish grey microtrichose, mesoscutum with scarcely distinct, rather darker vittae between rows of acrostichal and dorsocentral setae (dorsal view), all setae dark. Chaetotaxy: proepisternum with 1–3 setae on lower part and bare on upper part; prosternum bare; postpronotal lobe with 1 long and 5 shorter setae; acrostichals narrowly biserial, lacking on prescutellar depression; about 10 uniserial dorsocentrals ending in 3–4 prescutellars (about 0.20 mm long in middle of rows); 1 presutural intra-alar; 1 presutural supra-alar (0–1 additional seta between presutural intra-alar and supra-alar); 3 notopleurals (1–3 rather long setae on anterior part of notopleuron); 2–3 postsutural supra-alars; 1 postalar; 4 scutellars (with 0–2 additional fine setae); laterotergite with numerous brown setae. **Legs** yellow to brownish yellow, more basal parts (coxae, femora and tibiae) slightly lighter than apical parts. Legs entirely microtrichose and brown setose. Fore femur with minute anteroventral and posteroventral setae, covered with very short setose dorsally. Fore tibia short setose. Mid femur with sparse row of anteroventral setae nearly as long as femur width in basal part of femur and much shorter apically and denser row of posteroventral setae half as long as femur width in basal half of femur and somewhat longer in apical half. Mid tibia short setose dorsally and ventrally, with complete anteroventral row of spinules. Hind femur with irregular rows of anteroventral and posteroventral setae in basal half of femur nearly as long as (or slightly longer than) femur width; apically, they are slightly shorter, dorsal setae short. Hind tibia with scarcely differentiated (mostly posterodorsal) setae at most as long as tibia width, anteroventral row consisting of short spines, posteroventrals of equally short but seta-like; with rather short seta in posteroapical comb. Fore basitarsus slightly thickened (nearly as broad as fore tibia at apex), mid and fore basitarsi slender; all basitarsi short setose, fore basitarsus with several fine, longer ventral setae. **Wing** clear, stigma light brownish, veins brown; vein CuA+CuP slightly weakened in basal half, distinct in apical half toward wing margin. Costal seta absent or very short, axillary excision 90º. Measurements: M_2_/dm = 1.4–1.7, M_4_ ap/mp = 1.8–2.5, wl/ww = 2.6–2.8. Halter yellow, calypter yellow with brown fringes. **Abdomen** brown to yellowish brown (contrastingly lighter than thorax), entirely microtrichose and dark setose. Chaetotaxy: hind marginal setae almost as long as their corresponding segments, discal setae slightly shorter; dorsum of tergites with rather long setae. **Terminalia** as in Figure 8B,C; mostly yellowish brown. Epandrial lamella very short, subtriangular (lateral view), bearing several short, strong, black spines on apex and several long, fine setae on about middle. Cercus semiglobular, much larger than epandrium, with small finger like projection dorsally about base, pale tomentose apically. “Subcercal” process short and hidden under cercus. Phallus short.

**Female** (Figure 8D). Length: body 5.7–6.6 mm, wing 6.0–7.0 mm. Similar to male but with the following exceptions. Eyes broadly dichoptic, dorsalmost ommatidia slightly smaller than ventral ommatidia. Ocellar setae as long as frons. Frons 0.21 mm broad and 0.25 mm long, with 3–6 setae on each side (some of them may be very long and strong). Face as broad as frons and 0.20 mm long. Antennomere ratio = 12:9:39:11. Labrum slightly longer than head height. Thorax similarly coloured and setose as in male; 24–32 acrostichals, 14–16 dorsocentrals with tendency to be biserial, ending in 2–3 prescutellars (dorsocentrals about 0.20 mm long as in male, acrostichals slightly shorter). Legs similarly coloured as in male but differently setose. Fore leg as in male. Mid femur with short and sparse anteroventral setae, posteroventrals rather dense and slightly shorter than femur width, dorsal setae short. Mid tibia short setose, setae scarcely differentiated from setae. Hind femur with anteroventral and denser posteroventral setae nearly as long as femur width, anteriorly there is almost bare stripe. Hind tibia with dorsal setae (mostly in posterodorsal position) nearly as long as tibia width, ventral setae short and not spinose. Basal tarsomeres as in male. Wing broadened, dark brown (except small pale space between R_1_ and common Rs stem), stigma equally dark, veins dark brown, CuA+CuP almost uniformly sclerotised; axillary excision as in male. Measurements: M_2_/dm = 1.1–1.4, M_4_ ap/mp = 2.0–2.2, wl/ww = 2.1. Calypter and halter as in male. Abdomen brown to yellowish-brown, entirely grey microtrichose. Hind marginal setae on tergites 2–4 about half as long as their corresponding segment, on remaining segments they are shorter, discal setae shorter than marginals.

**Differential diagnosis:** *Rhamphomyia* (*P.*) *globulicauda* sp. nov. is a member of the *R.* (*P.*) *tipularia* complex (see also the discussion under *R. amurensis* sp. nov. and *R. epandriata* sp. nov.). Males of the new species described above may be easily distinguished according to the characteristic shape of male genitalia. Females are superficially very similar to *R. amurensis* sp. nov., however, they have densely setose legs, especially mid and hind femora covered posteroventrally with relatively dense setation.

**Distribution:** Russia (Magadanskaya Province, Yakutia).

**Dates of occurrence:** June.

### 3.9. Rhamphomyia (Pararhamphomyia) haladai sp. nov.

Zoobank link: urn:lsid:zoobank.org:act: F0C55B7A-F02A-4F50-8D72-D493C2887006

(Figure 9A,B)

**Type material: HOLOTYPE** ♂, labelled: Kazakhstan: Almaty prov., 8 km W of Saryzhaz [42°54′ N 79°36′ E], 1900 m, 42.910, 79.504, 6.vi.2016, J. Halada (CULSP).

**Diagnosis:** A relatively small species of *Rhamphomyia* (*Pararhamphomyia*) (body 3.2 mm) with entirely white to yellow setose thorax and abdomen; male pregenital segments unmodified.

**Etymology:** The species epithet, *haladai*, is a Latin genitive patronym to honour our colleague and expert insect collector, Mr. Jiří Halada (Třeboň), who kindly provided to one of us (M.B.) with very valuable materials.

**Figure 9:**
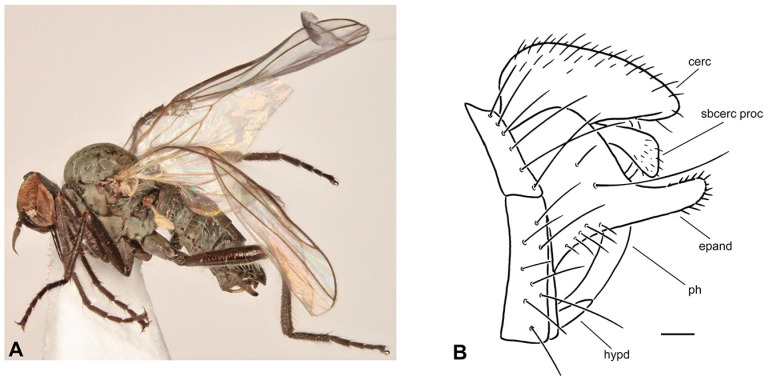
*Rhamphomyia* (*Pararhamphomyia*) *haladai* sp. nov., male holotype. (**A**) = Habitus, lateral view. (**B**) = Terminalia, lateral view. Abbreviations: cerc—cercus, epand—epandrial lamella, hypd—hypandrium, ph—phallus, sbcerc proc—subcercal process. Scale bar 0.1 mm.

**Description: Male** (Figure 9A). Length: body 3.2 mm, wing 3.9(?) mm. **Head** regions black to brownish black, light grey microtrichose. Eyes holoptic; upper ommatidia much enlarged. Frons brownish black, bare. Ocellar setae missing. Face 0.19 mm broad ventrally and 0.16 mm long, bare; clypeus lustrous in anterior part, posteriorly microtrichose. Occiput rather sparsely and long pale (whitish yellow) setose, longest setae up to 0.20 mm long, postocular row incomplete, confined to uppermost part. Antenna black, scape and pedicel rather short setose; antennomere ratio = 8:8:30:11. Labrum brown, shiny, 3/4 as long as head height; labella long, broad, long setose. Palpus brown, short, without prominent setae. Gena narrow, lustrous. **Thorax** black, light grey (slightly bluish grey) microtrichose; mesoscutum with two darker and more brownish vittae between rows of acrostichal and dorsocentral setae. All setae pale (whitish-yellow). Chaetotaxy: proepisternum with several setae in lower part and 3–4 setae in upper part; prosternum bare; postpronotal lobe with 1 strong, long and several shorter setae; acrostichals narrowly biserial, fine and long (about 7 setae per row); uniserial dorsocentrals (about 10 per row) even longer than acrostichals (longest setae up to 0.30 mm long), ending in 2 strong and very long prescutellars; 1 presutural intra-alar, 1 presutural supra-alar, 2 long and strong notopleurals (anterior part of notopleuron with only minute setulae), 2 supra-alars, postalar(s) missing; one pair of scutellars; laterotergite with several setae. **Legs** brown, mostly microtrichose (only hind femur anteriorly lustrous), pale and black setose; coxae concolorous with pleura, pale setose. Fore femur with short setae, anteroventrals and posteroventrals very fine, nearly 2/3 as long as femur width. Fore tibia with 1 anterodorsal seta twice as long as tibia width; remaining anterodorsal and posterodorsal setae fine, of different length and arrangement, at most slightly longer than tibia width. Mid femur with fine setae of subequal lengths anterodorsally to anteroventrally (nearly as long as femur width); row of additional spine-like anteroventral short setae; fine posteroventrals similar to those on fore femur. Mid tibia with 3–4 long, strong, anterodorsals including preapical (longest setae up to 0.35 mm long—longer than basitarsus); 2 shorter posterodorsals; spine-like ventral setae arranged in two irregular rows (shorter than tibia width). Hind femur robust but not much swollen; covered with rather dense, irregularly arranged setae (nearly as long as femur width); ventral surface microtrichose. Hind tibia slightly curved in about basal third, slightly widening towards apex; covered with irregularly arranged fine setae dorsally and ventrally (slightly longer than tibia width); 5–6 slightly longer and thicker setae dorsally; 1 short seta in posteroapical comb. Fore basitarsus slender, with conspicuous subapical circlet of setae, bearing fine apically curved setae ventrally; mid and hind basitarsi equally slender, without conspicuous setation. **Wing** clear, stigma pale, indistinct, veins brown to yellowish brown. Vein CuA+CuP incomplete and weakened even in basal part. Basal costal seta apparently present and strong but missing on both wings. Axillary excision slightly obtuse. Measurements: M_2_/dm = 1.6, M_4_ ap/mp = 2.5(?), wl/ww = 2.8(?) (wings shrunken). Halter yellow with brownish base; calypter yellow with yellow fringes. **Abdomen** black, light grey microtrichose, with distinct brown tinge viewed dorsally. Chaetotaxy: setae all pale (whitish-yellow); lateral posteromarginal setae long, up to 0.25 mm long, rather dense on basal five segments, slightly shorter and sparser on remaining segments; dorsum of tergites with shorter setae; segment 8 with conspicuously long setae (especially tergite). **Hypopygium** (Figure 9B, not dissected) small; epandrium and cerci concolorous with abdominal tergites. Epandrial lamella broad basally and strongly narrowed (almost claw-like) apically (lateral view); basal broadened part with 1 strong, long, white seta; apical narrow part with short spines on both sides. Hypandrium short, shiny, bare. Cercus with dorsal part broad; ventral part narrower and slightly widening apically, still broader than narrow part of epandrium.

**Female.** Unknown.

**Differential diagnosis:** There are only two described species of Palaearctic *Rhamphomyia* (*Pararhamphomyia*) with uniserial dorsocentrals and all setae on mesoscutum and abdomen whitish yellow besides the above-described species, *R. spinicauda* sp. nov. However, *R*. *haladai* sp. nov. has unmodified male pregenital segments. Apically strongly narrowed epandrium forming “claw-like” structure resembles *R. unguiculata,* but the latter is quite a different species.

**Distribution:** Kazakhstan.

### 3.10. Rhamphomyia (Pararhamphomyia) indigirka sp. nov.

Zoobank link: urn:lsid:zoobank.org:act: B69CE657-18A0-4209-A58E-814438FB7A00

(Figure 10A,B)

**Type material: HOLOTYPE** ♂, labelled: [in Cyrillic] Russia [Sakha-Yakutia], r. [=river] Indigirka/ust. r. [=mouth of river] Injali [65°14′ N 143°08′ E]/24.vi.1976, V. Kovalev//listvennicznik s ernikom i mchom [=larch forest with dwarf birch and moss], 500–600 m (ZMMU).

**Diagnosis:** Small species (wing about 3.5 mm) of *Rhamphomyia* (*Pararhamphomyia*) with black setose body, bare proepisternal depression, biserial dorsocentral setae, clear to slightly milky white on base wings, brown halter, short posteroventral setae on hind femur.

**Figure 10:**
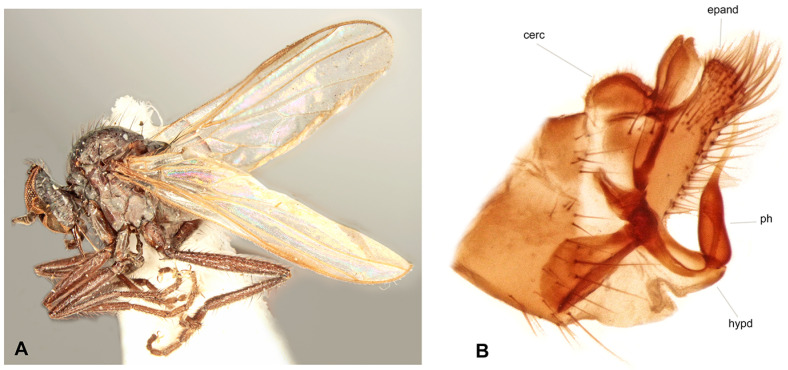
*Rhamphomyia* (*Pararhamphomyia*) *indigirka* sp. nov., male holotype. (**A**) = Habitus (dissected), lateral view. (**B**) = Terminalia, lateral view. Abbreviations: cerc—cercus, epand—epandrial lamella, hypd—hypandrium, ph—phallus.

**Etymology:** The species is named after the Indigirka river.

**Description: Male** (Figure 10A). Length: body 3.1 mm (without genitalia), wing 3.3 mm. **Head** regions black to brownish black, light grey microtrichose. Eyes holoptic, upper ommatidia much enlarged. Frons bare. Ocellar setae missing, ocellar triangle with 4 additional setae along hind margin. Face 0.15 mm broad ventrally and 0.17 mm long, bare. Occiput black setose, postocular row complete but irregular. Antenna with scape and pedicel brown, postpedicel and stylus black; both basal antennomeres short setose (longest setae 0.10 mm long); antennomere ratio = 6:7:27:10. Labrum brown, shiny, half as long as head height. Palpus brown, bearing a few setae. Gena narrow, microtrichose; clypeus microtrichose. **Thorax** brownish black, almost light grey microtrichose, without vittae. All setae black. Chaetotaxy: proepisternum wit 5–6 long setae on lower part and bare on upper part; prosternum bare; 1 long postpronotal and several shorter setae; about 16 biserial acrostichals; 11 irregularly biserial dorsocentrals, ending in 2 prescutellars; acrostichals and dorsocentrals moderately strong, about 0.20 mm long in middle of rows; 1 presutural intra-alar; 1 presutural supra-alar (with 4–6 rather long setae outside of dorsocentrals in presutural area); 3 notopleurals, (3–4 rather long setae on anterior part of notopleuron); 2 postsutural supra-alars (with 2 setae on prealar area); 1 long and 1 short postalar; 4 scutellars (outer pair only slightly shorter than inner pair); laterotergite with numerous setae. **Legs** (including coxae) brown, microtrichose, black setose. Fore femur with sparse rows of anteroventral and posteroventral short setae (as long as femur width), dorsal setae short. Fore tibia with long posterodorsal setae in basal third (twice as long as tibia width), shorter setae apically and short ventral setae. Mid femur with row of sparse, short, anteroventral setae, posteroventral setae in apical third (about as long as femur width) and short dorsal setae. Mid tibia with 2 long anterodorsal setae in basal third (besides 1 preapical seta; twice as long as tibia width), covered with uniform, moderately long setae posterodorsally (slightly longer than tibia width), bearing complete row of posteroventral spinose setae and finer anteroventral setae apically. Hind femur with row of anteroventral setae, which are short in basal half and get longer toward apex (in apical third nearly 1.5× as long as femur width), shorter posteroventral setae (except 1 posteroventral subapical seta) and mostly short dorsal setae (longer only near base of femur). Hind tibia with 5–6 irregularly arranged, rather long setae dorsally (about 1.5 times as long as tibia width) and anteroventral row of spinose setae (longest setae slightly longer than tibia width); long seta in posteroapical comb. Fore basitarsus slender, with dorsal setae only slightly longer than its diameter; mid basitarsus slender, dorsally covered with setae more than twice as long as its diameter, anteriorly and ventrally with several spine-like setae; hind basitarsus rather slender and short setose. **Wing** clear, slightly milky white on base, stigma brownish, veins yellowish or hyaline, vein CuA+CuP absent in apical half. Costal seta present, axillary excision right. Measurements: M_2_/dm = 1.3, M_4_ ap/mp = 2.0, wl/ww = 2.5–2.6. Halter brown; calypter brownish yellow, with dark fringes. **Abdomen** brownish black, grey microtrichose, covered with black setae. Chaetotaxy: posteromarginal setae on sides of tergites subequally long as segments, discal setae only slightly shorter than marginals; dorsum of tergites with rather long setae. **Hypopygium** as in Figure 10B; epandrium and cerci concolourous with abdominal tergites, black setose. Epandrial lamella simple, without dorsal tuft of setae, bearing numerous short setae over lower margin and apically. Hypandrium subtriangular (ventral view), bare. Cercus bilobate (deeply concave dorsally), covered with fine setulae. Phallus with long thickened portion beyond basal curvature, thin apical part short, slightly bowed.

**Female.** Unknown (see remarks under Differential diagnosis).

**Differential diagnosis:** *Rhamphomyia* (*P.*) *indigirka* sp. nov. belongs to the *R. pusilla* complex of species and it is most allied to *R. pusilla* (Zetterstedt) and *R. hoeli* Frey*. Rhamphomyia indigirka* sp. nov. differs from *R. pusilla* in male sex by lighter wings (they are brownish in *R. pusilla* even along base), bare proepisternal depression and short posteroventral setae on hind femur (subequally long and numerous as anteroventrals in *R. pusilla*), and from *R. hoeli* it differs by genitalia (in *R. hoeli* cranial lobe of cercus elongated, only slightly shorter than epandrium and “subcercal” process is shiny apically). There are also slight differences between male genitalia of *R. pusilla* and *R. indigirka* sp. nov.: the “subcercal” process in the former species is much broader and longer setose than in the latter species and the narrow part of the phallus is bent in a sharper (almost right) angle. The female of the new species remains unknown; however, there are several females in ZMMU belonging highly probably to either *R. indigirka* sp. nov. or to *R. spiraliseta* sp. nov. Both have multiserial dorsocentral setae, completely dark setose body, legs not pennate, dark halter and microtrichose mesoscutum. As far as we know, only two Palaearctic species sharing these characters with presumed *R. indigirka* sp. nov. and/or *R. spiraliseta* sp. nov. have incomplete vein CuA+CuP: *R. filicauda* Henriksen & Lundbeck and *R. pusilla*. However, *R. filicauda* has densely setose mesonotum (more than 10 scutellar setae and great number of notopleurals) and *R. pusilla* has setose proepisternal depression. There are at least two Nearctic species with similar genitalia as *R. pusilla*, *R. spiraliseta* sp. nov. or *R. indigirka* sp. nov., but both have deformed legs.

**Distribution:** Russia (Yakutia).

**Dates of occurrence:** June.

### 3.11. Rhamphomyia (Pararhamphomyia) krivosheinae sp. nov.

Zoobank link: urn:lsid:zoobank.org:act: 078DA1E0-0DE0-4E20-9375-EBDD03FCCAEE

(Figure 11A,B)

**Type material: HOLOTYPE** ♂, labelled: [Russia], Amurskaya obl. [=province], g. [=town] Zeya [53°44′ N 127°15′ E], 29.vi.1982, M. Krivosheina (ZMMU). **PARATYPES: RUSSIA, Amurskaya Prov.:** same locality as holotype, 25.vi.1982, leg. A. Ozerov (1 ♂, ZMMU); 30.vi.1978, leg. A. Shatalkin (1 ♂, ZMMU).

**Figure 11:**
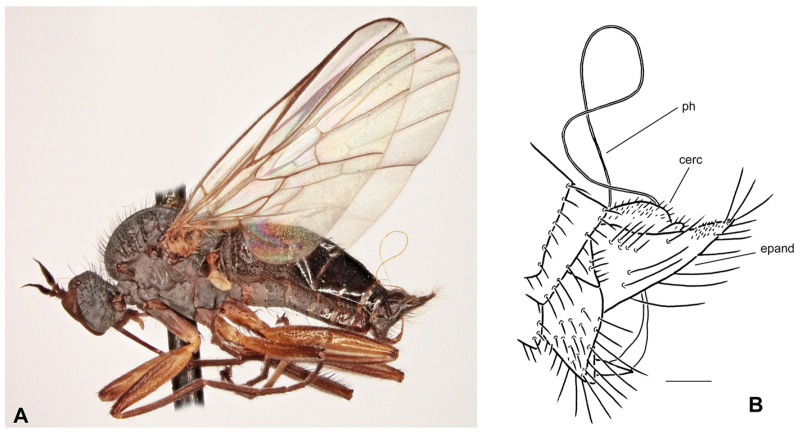
*Rhamphomyia* (*Pararhamphomyia*) *krivosheinae* sp. nov., male holotype. (**A**) = Habitus, lateral view. (**B**) = Terminalia, lateral view. Abbreviations: cerc—cercus, epand—epandrial lamella, ph—phallus. Scale bar 0.1 mm.

**Diagnosis:** Medium-sized species of *Rhamphomyia* (*Pararhamphomyia*) (body about 4.5 mm) with black setose body, 2–3-serial dorsocentral setae, light grey microtrichose mesoscutum, yellow halteres and extensively yellow legs. Male: phallus very long and thin.

**Etymology:** The species is named in honour of Russian dipterist Marina Krivosheina (Moscow), the collector of the holotype.

**Description: Male** (Figure 11A). Length: body 4.5 mm (without genitalia), wing 4.3 mm. **Head** regions black, light grey microtrichose. Eyes broadly dichoptic, upper ommatidia slightly smaller than lower ommatidia. Frons 0.20 mm broad and 0.28 mm long, with rows of 4–5 rather long setae on each side. Ocellar setae black, strong, about as long as frons; ocellar triangle without additional setae. Face equally broad as frons and 0.24 mm long, bare. Occiput covered with fairly strong and short black setae; postocular row incomplete, absent in middle part. Antenna blackish brown, scape and pedicel short setose; antennomere ratio = 15:10:36:12. Labrum brown, shiny, slightly longer than head height. Palpus brown, short, bearing a few setae. Gena narrow, microtrichose. **Thorax** black, light grey microtrichose, in dorsal view without vittae, in posterior view with indefinite darker vittae down rows of acrostichal and dorsocentral setae; black setose. Chaetotaxy: proepisternum with 3 setae on lower part and bare on upper part; prosternum bare; postpronotal lobe with 1–2 long and numerous additional shorter setae of subequal length and robustness; more than 30 biserial acrostichals; about same number of irregularly 2–3 serial dorsocentrals, ending in irregularly arranged 3–4 prescutellars; acrostichals and dorsocentrals moderately strong and about 0.20 mm long in middle of rows, several setae present also on prescutellar depression; sides of mesoscutum in presutural area outside of dorsocentrals densely covered with similar setae as dorsocentrals, presutural intra-alar seta not differentiated from surrounding setae and presutural supra-alar seta only slightly stronger; 4–5 notopleurals scarcely differentiated from numerous surrounding setae on anterior part of notopleuron; 1 long and strong postsutural supra-alar (with 4 setae on prealar area and 2 additional setae); 1 long and strong postalar (with several additional setae); 4 scutellars (lateral pair slightly shorter than apical pair); laterotergite with numerous setae. **Legs** extensively yellow; coxae brownish yellow about base and yellow more distally, dorsum of fore femur (and also parts of mid and hind femora), tibiae and tarsi brownish yellow; microtrichose, covered with dark setae. Fore leg short setose. Fore femur lacking setae ventrally, with microtrichosity. Mid femur almost bare anteroventrally; posteroventrally with irregular and dense row of long setae in middle part of femur (nearly 1.5× as long as femur width); dorsal setae short. Mid tibia with short dorsal setae and dense, short, ventral pubescence. Hind leg similarly setose as mid leg; hind tibia with 1 long seta in posteroapical comb. Basitarsus of all legs slender and short setose, without ventral spines. **Wing** clear, stigma hyaline, veins brown; vein CuA+CuP depigmented in apical part. Basal costal seta present. Axillary excision slightly obtuse. Measurements: M_2_/dm = 1.2–1.3, M_4_ ap/mp = 1.8–1.9, wl/ww = 2.1–2.2. Halter yellow; calypter yellow, with pale fringes. **Abdomen** blackish brown, brownish grey microtrichose, tergites 4–7 and sternites 5–7 mostly shiny, dark setose. Chaetotaxy: posteromarginal setae on sides of tergites nearly 2/3 as long as their corresponding segment, discal setae shorter; dorsum of tergites slightly shorter setose. **Hypopygium** as in Figure 11B (not dissected); epandrium and cerci concolorous with abdominal tergites but denser microtrichose, epandrium with pale setae. Epandrial lamella subtriangular (lateral view). Hypandrium subtriangular (ventral view), bare. Cercus broadly rounded at apex; “subcercal” process narrow. Phallus mostly very long and thin (thickened basally).

**Female.** Length of body: 4.2–4.6 mm, wing 4.0–4.4 mm. Similar to male, except as follows. Ratio of antennomeres = 12:9:35:11. Labrum 1.4 times as long as head height, slightly curved. Thorax with setae slightly shorter (both acrostichals and dorsocentrals about 0.15 mm long) and less numerous. Legs slightly darker than in male, only basal half of all femora clear yellow, other parts of legs yellowish-brown. Fore femur short setose, anteroventrals and posteroventrals present. Fore tibia short setose. Mid femur short setose, anteroventrals short, posteroventrals somewhat longer (nearly half as long as femur width) and stronger. Mid tibia short setose, 1–2 anterodorsals and anteroventrals differentiated. Hind femur short setose, both anteroventrals and posteroventrals present. Hind tibia short setose, anterodorsals and posterodorsal slightly differentiated but shorter than tibia width. Basitarsi slender and short setose, with short ventral spines. Wing as in male. Measurements: M_2_/dm = 1.4–1.5, M_4_ ap/mp = 1.9, wl/ww = 2.6–2.7. Abdomen blackish brown, light grey microtrichose; posteromarginal setae on sides of abdomen short, about 0.16 mm long, discal setae half as long.

**Differential diagnosis:** *Rhamphomyia* (*P.*) *krivosheinae* sp. nov. belongs to the *R. praestans* complex of species (see also discussion under *R. angustitibia* sp. nov.). However, it differs from other species of this complex in having at least partly yellow legs. Besides colour characters, the new species differs from *R. praestans* Frey in having longer setose hind tibia (individual setae nearly 0.15 mm long whereas only half as long in *R. praestans*), hind femur with anteroventral setae, which do not spread behind basal third of femur (but they occupy more than half length of the femur in *R. praestans*) and slight differences also in male terminalia (e.g., epandrium bears preapical dorsal bump in *R. praestans*, which is absent in *R. krivosheinae* sp. nov.)

**Distribution:** Russia (Amurskaya Province).

**Dates of occurrence:** June.

### 3.12. Rhamphomyia (Pararhamphomyia) morgunovka sp. nov.

Zoobank link: urn:lsid:zoobank.org:act: 6DEBBD90-2A56-4105-8170-5B2DCA620F16

(Figure 12A–C)

**Type material: HOLOTYPE, ♂** labelled: [**Turkmenistan**], Morgunovka [now Serkhetli, 35°17′ N 62°23′ E], env. Kushka, 14.iv.1976, leg. V. Kovalev (terminalia dissected and attached in microvial, ZMMU).

**Diagnosis:** Rather small species of *Rhamphomyia* (*Pararhamphomyia*) (body about 3.5 mm) with black setose body, grey mesoscutum, uniserial dorsocentral setae, dark legs and pale halteres; epandrium elongated at apex, which is incurved and slightly downcurved; cercus with oblong “subcercal” process.

**Etymology:** The species is named after the type locality.

**Figure 12:**
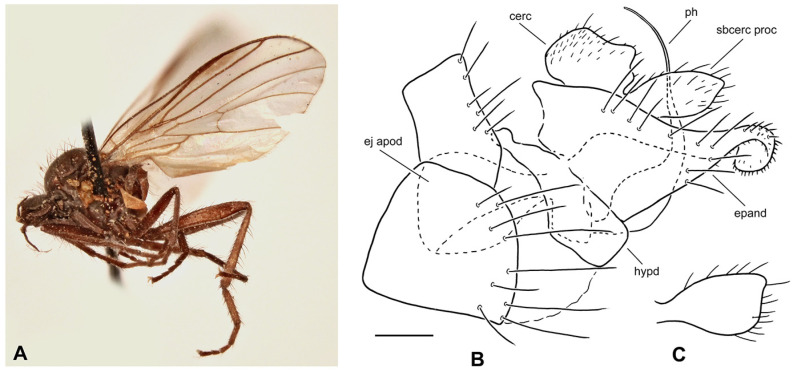
*Rhamphomyia* (*Pararhamphomyia*) *morgunovka* sp. nov., male holotype. (**A**) = Habitus (dissected). (**B**) = Terminalia, lateral view. (**C**) = Subcercal process. Abbreviations: cerc—cercus, epand—epandrial lamella, hypd—hypandrium, ej apod—ejaculatory apodeme, ph—phallus, sbcerc proc—subcercal process. Scale bar 0.1 mm.

**Description: Male** (Figure 12A). Length: body 3.2 mm (without genitalia), wing 3.5 mm. **Head** regions black, grey microtrichose. Eyes holoptic, upper ommatidia much enlarged. Frons bare. Ocellar setae black, half as long as frons, no additional setae on ocellar triangle. Face about 0.15 mm broad ventrally and 0.10? mm long, bare; clypeus microtrichose. Occiput rather sparsely black setose, postocular row present only in its dorsal part. Antenna with scape and pedicel brown, postpedicel and stylus black, scape and pedicel short setose (longest setae about 0.08 mm long); antennomere ratio = 7:6:25:9. Labrum brown, shiny, only slightly shorter than head height. Palpus brown, short, bearing a few rather long setae (longest setae about 0.20 mm long). Gena narrow, microtrichose. **Thorax** brownish black, grey microtrichose, mesoscutum without distinct vittae. All setae black. Chaetotaxy: proepisternum with 1–2 long setae on lower part and bare on upper part; prosternum bare; postpronotal lobe with 1 long and 4 shorter setae; about 9 narrowly biserial acrostichals; 9 biserial to nearly uniserial dorsocentrals (only 2 setae standing outside row anteriorly) moderately strong and slightly longer than acrostichals (0.18 mm long in middle of row), ending in 1 longer prescutellar seta; 1 presutural intra-alar and 1 presutural supra-alar (with 1–2 additional fine setae); 3 notopleurals (lower seta seta situated unusually cranially; 4 rather long fine setae on anterior part of notopleuron); 1 supra-alar and 4 additional fine setae on prealar area; 1 postalar; 4 almost equally long scutellars; laterotergite with numerous setae. **Legs**, including coxae, brown, microtrichose, black setose. Fore femur with complete rows of anteroventral and posteroventral setae (slightly longer than femur width). Fore tibia with almost uniform setation dorsally (as long as or slightly longer than tibia width), ventral setae short. Mid femur with row of anteroventral and anterior short setae (slightly shorter than femur width); longer posteroventral setae (in apical third nearly 1.5× as long as femur width). Mid tibia with 3 anterodorsal and 5–6 posterodorsal moderately long setae (about twice as long as width of rather slender tibia); two almost regular rows of sparse, spinose, anteroventral and posteroventral setae in apical 2/3 (shorter than tibia width). Hind femur with anteroventral row of setae in apical third (slightly longer than femur width); short posteroventral setae (only just before tip 3 fine setae shorter than femur width); short dorsal setae. Hind tibia with 4–5 pairs of setae dorsally (nearly 1.5 times as long as tibia width); anteroventral row of spinose setae (slightly shorter than tibia width), short posteroventral setae; 1 long seta in posteroapical comb. Basitarsus of all legs slender, short setose, with short ventral spines. **Wing** clear, stigma brownish, veins yellowish-brown, CuA+CuP absent in apical third. Costal seta present, axillary excision slightly obtuse. Measurements: M_2_/dm = 1.5–1.6, M_4_ ap/mp = 2.3–2.4, wl/ww = 2.6?. Halter yellow, calypter brownish with dark fringes. **Abdomen** black, light grey microtrichose, dark setose. Chaetotaxy: posteromarginal setae on sides of tergite 2 nearly as long as segment, on segments 3–4 half as long, on segments 5–6 1/3 as long as their corresponding segment; discal setae shorter than marginals; dorsum of tergites shorter setose. **Terminalia** (Figure 12B,C): epandrium and cerci concolourous with abdominal tergites. Epandrial lamella elongated at apex, which is incurved and slightly downcurved. Cercus deeply concave dorsally; with oblong “subcercal” process. Phallus thickened at about basal 1/3, remaining portion slender.

**Female.** Unknown.

**Differential diagnosis:** *Rhamphomyia* (*P.*) *morgunovka* sp. nov. belongs to the *R. albipennis* complex of species. The new species differs from other species of this complex (*R. unguiculata* Frey*, R. albipennis* (Fallén)*, R. murina* Collin) primarily in the shape of male terminalia. *Rhamphomyia unguiculata* has a long claw-like process at the apex of the epandrium; *R. albipennis*—“beak-like” opened tip of “subcercal” process and *R. murina* is densely setose species with pointed “subcercal” process and short postpedicel. On the other hand, the “subcercal” process of *R. morgunovka* sp. nov. is somewhat similar to that of *R. pusilla* (Zetterstedt). Because dorsocentral setae have tendency to be uniserial, this new species may be confused with *R. acuticauda* sp. nov. or *R. hoeli* Frey (because all these species have dark legs, pale halter, entirely black setose body, simple legs and phallus as well as grey microtrichose mesoscutum). The best differential characters are in the shape of terminalia (see also discussion under *R. acuticauda* sp. nov.). The female of the new species remains unknown.

**Distribution:** Turkmenistan.

**Dates of occurrence:** April.

### 3.13. Rhamphomyia (Pararhamphomyia) norgensis sp. nov.

Zoobank link: urn:lsid:zoobank.org:act: 1F81B0ED-6905-46B9-AB12-7E4A860A8541

(Figure 13A–C)

**Type material: HOLOTYPE ♂**, labelled: N [=**NORWAY**]: Vågåmo [61°53′ N, 9°6′ E], 11.vii.1953 [no collector’s name] (MZLU). **PARATYPES: NORWAY:** same data as holotype (1 ♀, CULSP). **RUSSIA. Altay Rep.:** Ust-Koksinskiy District, 10 km S Katanda, 19.vii.1983, A. Barkalov (1 ♂, ZISP); **Amurskaya Prov.:** town Zeya, 24.vii.1979, A. Shatalkin leg. (1 ♂, ZMMU); same locality, 7.vii.1978, A. Shatalkin (1 ♂, ZISP). **Buryatia Rep.:** env. Mondy, Sayany, Tunkinskie Goltsy Range, 1600–1800 m, swamp, 19.vii.1965, Gorodkov (1 ♂, ZISP). **Sakha (Yakutia) Rep.:** Amginsk-Yakutsk road, 14.viii.1925, L. Bianki (1 ♂, ZISP).

**Diagnosis:** Medium-sized species (body about 4.5 mm) of *Rhamphomyia* (*Pararhamphomyia*) with extensively shiny mesoscutum, pale setose abdomen and yellow halter. Male: terminalia greatly modified, asymmetrical. Female: mid femur and tibia as well as hind femur and tibia broadly pennate.

**Etymology:** The species is named after the holotype country of origin.

**Figure 13:**
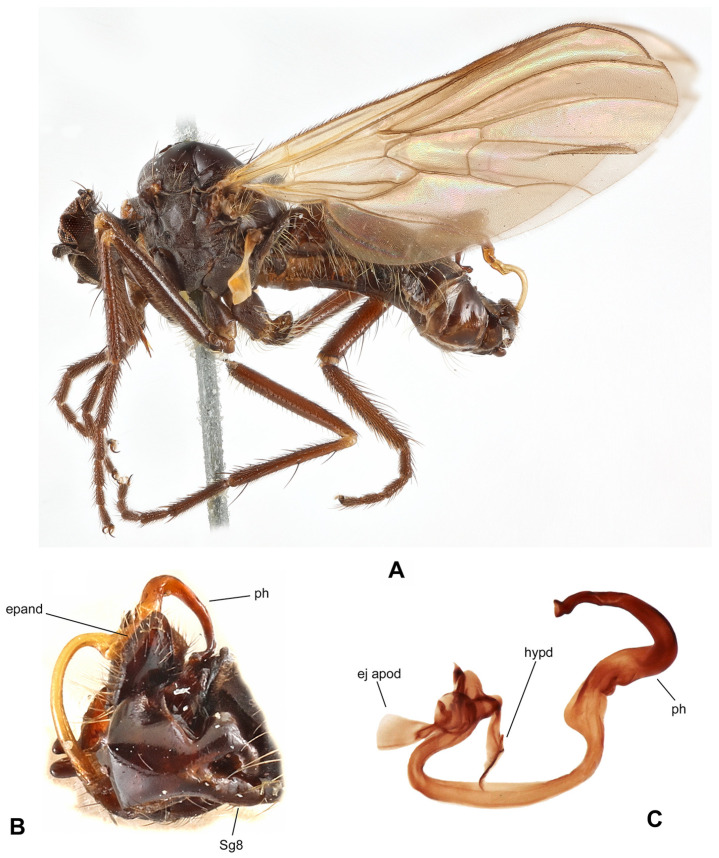
*Rhamphomyia* (*Pararhamphomyia*) *norgensis* sp. nov., male. (**A**) Habitus, lateral view. (**B**) = Terminalia, right lateral view. (**C**) = Hypandrium and phallus lateral view. Abbreviations: epand—epandrial lamella, hypd—hypandrium, ej apod—ejaculatory apodeme, ph—phallus, Sg8—abdominal segment 8.

**Description: Male** (Figure 13A). Length: body 4.2–4.4 mm, wing 4.8 mm. **Head** regions brownish black to brown, grey microtrichose (except noted). Eyes holoptic, upper ommatidia enlarged. Frons represented by two small subtriangular spaces just below ocellar triangle and above antennae, bare. Ocellar setae dark, scarcely 1/3 as long as frons, ocellar triangle without additional setae. Face somewhat subshiny below, about 0.20 mm broad below and 0,35 mm long, bare; clypeus mostly microtrichose. Occiput rather light grey microtrichose, sparsely and short, mostly black setose on upper part and pale setose on lower part. Antenna with scape and pedicel brown, postpedicel and stylus black, longest setae on scape and pedicel about 0.10 mm long; antennomere ratio = 14:9:30:13. Labrum brown, shiny, slightly shorter than head height. Palpus brown, short; short, fine setose. Gena narrow. **Thorax** brown in ground-colour, mostly greyish microtrichose; disc of mesoscutum shiny; postpronotal lobe, margins of mesoscutum, prescutellar depression and scutellum faintly microtrichose. Large thoracic setae mostly black (postpronotal and anterior notopleural setae usually pale, sometimes black); smaller setae pale to white (including setae of prothoracic sclerites, acrostichals and anterior dorsocentrals as well as fine setae situated on supra-alar face and on anterior part of notopleuron). Chaetotaxy: prosternum bare; proepisternum with tuft of 6–10 setae in lower part, bare or with 1 seta on upper part; postpronotal lobe with 1 strong, long and several much shorter fine setae; acrostichals short and very fine, irregularly biserial, 10–12 setae per row, lacking on prescutellar depression; presutural dorsocentrals irregularly biserial, postsuturals uniserial, ending in 3 long prescutellars, dorsocentrals about 0.15 mm long, acrostichals slightly shorter; 1 presutural intra-alar seta very scarcely differentiated, 1 strong presutural supra-alar, 3 notopleurals (several very short and fine setae on anterior part of notopleuron); 1 strong postsutural supra-alar and 2–4 very short fine setae on prealar area; 1 long and 1 short postalar; 4 scutellars (apical pair longer; sometimes additional short fine setae present); laterotergite with numerous pale setae. **Legs** brown, femora and tibiae mostly shiny and black setose, femora with some pale to yellowish setae closer to base. Coxae concolorous with pleura, microtrichose, pale setose. Fore femur not pubescent ventrally; with very short setae, both anteroventrals and posteroventrals at most 1/3 as long as femur width. Fore tibia with posterodorsal setation about as long as tibia width (setae scarcely differentiated), anterodorsals even shorter and ventral setae very short. Mid femur whitish tomentose ventrally; with row of mostly very short anteroventral setae (somewhat longer near base); setae of posteroventral row contrastingly longer (even near base), longest setae as long as femur width. Mid tibia with 2 irregular rows of ventral setae about as long as tibia width; 3 very long anterodorsal setae (basal seta somewhat shorter), other dorsal setation short; 1 very short seta in posteroapical comb. Hind femur slender; whitish tomentose ventrally; with row of fine anteroventral setae, which are distinct in about basal third (longest setae nearly as long as femur width) but inconspicuous in remaining portion (except a few preapicals); posteroventrals arranged similarly; dorsal setation short except a few fine anterodorsals. Hind tibia slightly swollen (about as wide as femur); with 1 submedian anterodorsal and 7–8 posterodorsal setae slightly longer than tibia width; ventral setae short. Fore and mid basitarsi slender; hind basitarsus slightly thickened at base and narrowed toward apex; with short but distinct spine-like setae ventrally, otherwise short setose, except hind basitarsus, which bears several setae dorsally somewhat longer than its diameter. **Wing** faintly brownish infuscate, pterostigma slightly darker, veins yellowish-brown; CuA+CuP complete (sometimes slightly weakened in middle). Costal seta present. Axillary excision 90º to slightly less. Measurements: M_2_/dm = 1.5, M_4_ ap/mp = 1.5, wl/ww = 3.3. Halter yellow; calypter yellow with pale fringes. **Abdomen** brown, pale setose; tergites 1–6 faintly microtrichose dorsally and shiny laterally; visible portion tergite 7 and tergite 8 shiny; sternites 1–5 shiny. Chaetotaxy: posteromarginal setae on sides of tergites 2–5 sparse but as long as their corresponding segment (discal setae shorter), tergite 6 contrastingly bare; dorsum of tergites with shorter setae. Pregenital segments 6–8 modified, particularly segments 7 and 8 greatly deformed; segment 8 with two small outgrowths on left side and two large sack-like swellings on right side (Figure 13B). **Hypopygium** (Figure 13B) rather small, somewhat retracted into segment 8, slightly rotated to left; epandrial lamella concolorous with abdominal tergites, cercus somewhat paler, both shiny; epandrial lamella with numerous, long, pale setae over lower margin; cercus with dark setulae. Epandrial lamella subtriangular (lateral view). Hypandrium reduced to lateral arms, membraneous ventrally. Cercus rather elongate oval, without additional projections. Phallus as in Figure 13C, somewhat asymmetrical, rather W-shaped.

**Female.** Length: body 5.5 mm, wing 5.2 mm. Similar to male, except as follows. Eyes broadly dichoptic, facets in dorsalmost part of eye smaller than ventral ones. Frons shiny in dorsal lateral corner, with 1–2 small setae, about 0.30 mm long and 0.20? mm broad. Ocellar setae strong, half as long as frons, ocellar triangle with two additional setae. Occiput sparsely covered with rather strong black setae dorsally, postocular row incomplete. Antennomere ratio = 20:9:33:15. Labrum as long as head height. Fore leg as in male, but fore tibia with slightly shorter setae. Mid femur and tibia as well as hind femur and tibia broadly pennate; this pennation is slightly less flattened on mid tibia ventrally and rather short in both basal and proximal third of hind tibia ventrally. Basal tarsomeres of all legs as in male. Wing pale brownish yellow, otherwise as in male; M_2_/dm = 1.5–1.6, M_4_ ap/mp = 1.5–1.6, wl/ww = 3.1; calypter brownish yellow. Abdominal tergites 2–8 shiny brown, with faint microtrichosity on basal part of dorsum, sternites 1–7 shiny or with very faint microtrichosity on some parts. Setae all pale. Posteromarginal setae on sides of tergites 2–3 about 1/2 as long as their corresponding segments (discal setae shorter), those on tergite 4 about 1/3 as long as segment, shorter on remaining tergites.

**Differential diagnosis:** *Rhamphomyia* (*Pararhamphomyia*) *norgensis* sp. nov. is superficially close to *R. tenuiterfilata* Becker, sharing a combination of the following characters: at least disc of mesoscutum shiny, devoid of microtrichiae, white setose abdomen and yellow halter. However, the male of *R. tenuiterfilata* has quite different terminalia with unmodified pregenital segments and the female has legs without pennation. *Rhamphomyia norgensis* sp. nov. is undoubtedly allied to *R. araneipes* Frey and *R. deformicauda* Saigusa, having very similar curiously asymmetrical and greatly deformed terminalia. However, both latter species are completely black setose besides some other differences. The allied species with similarly distorted phallus and modified pregenital segments occur also in the Nearctic region (at least three undescribed species).

**Distribution:** Norway, Russia (Altay Republic, Amurskaya Prov., Buryatia, Yakutia).

**Dates of occurrence:** July–August.

### 3.14. Rhamphomyia (Pararhamphomyia) nudifemorata sp. nov.

Zoobank link: urn:lsid:zoobank.org:act: D18F771B-7D51-4BCA-A838-8B6B5ED1C547

(Figure 14A,B)

**Type material: HOLOTYPE ♂**, labelled: [in Cyrillic, **RUSSIA**], Amursk. obl. [=Amurslaya oblast’, province], g. [=gorod, town] Zeja [53°44′ N 127°15′ E], 5.vii.1978/A. Shatalkin (ZMMU). **PARATYPE: RUSSIA. Amurskaya Prov.:** same locality as holotype, 9.vii. 1978, A. Shatalkin (1 ♂, CULSP).

**Diagnosis:** Relatively small species (body about 3.5) of *Rhamphomyia* (*Pararhamphomyia*) with yellow legs, virtually bare femora on anteroventral side and dichoptic eyes in both sexes.

**Etymology:** The species is named after the peculiarly bare femora.

**Figure 14:**
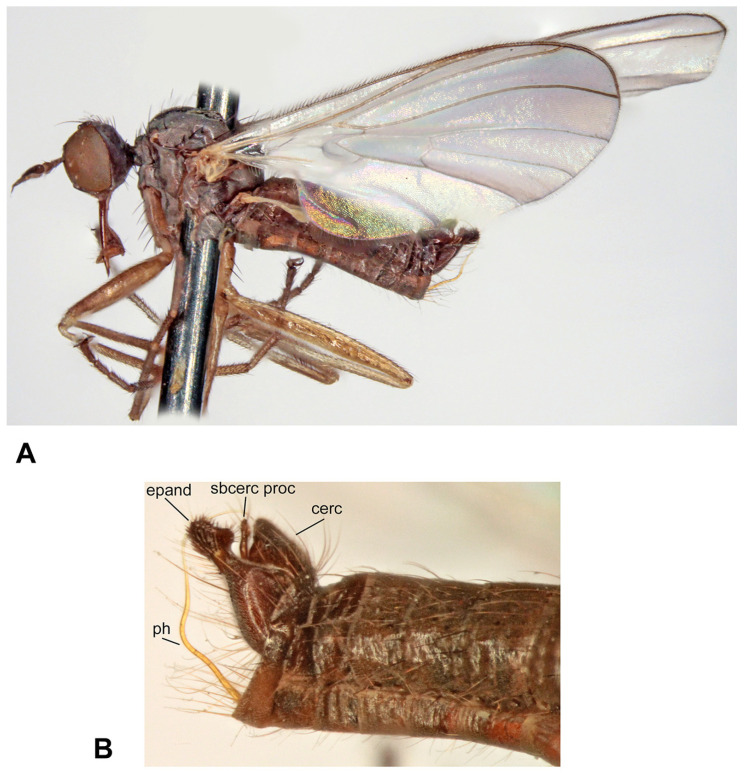
*Rhamphomyia* (*Pararhamphomyia*) *nudifemorata* sp. nov., male holotype. (**A**) = Habitus, lateral view. (**B**) = Postabdomen, lateral view. Abbreviations: cerc—cercus, epand—epandrial lamella, ph—phallus, sbcerc proc—subcercal process.

**Description: Male** (Figure 14A). Length: body 3.0 mm (without genitalia), wing 3.6 mm. **Head** regions brownish black to brown, light grey microtrichose. Eyes dichoptic, ommatidia of subequal. Frons 0.05 mm broad in narrowest place and 0.21 mm long; with 2 short setae ventrally. Ocellar setae black, as long as frons, ocellar triangle with several additional setae in posterior part. Face about 0.10 mm broad ventrally and 0.20 mm long, bare; clypeus microtrichose. Occiput sparsely and short black setose, setae almost arranged in two rows, postocular row incomplete in middle. Antenna brown, scape and pedicel rather short setose; antennomere ratio = 10:7:30:6. Labrum brown, shiny, as long as head height. Palpus brown, short, bearing a few setae. Gena narrow, microtrichose. **Thorax** brownish black, light grey microtrichose; mesoscutum with scarcely distinct darker vittae visible from some angles of view. All setae black. Chaetotaxy: proepisternum with 1 seta on lower part and bare on upper part; prosternum bare; postpronotal lobe with 1 long and 2 much shorter setae; only 2 acrostichals visible on anterior half of mesoscutum; 5? uniserial dorsocentrals slightly longer than acrostichals (0.15 mm long), ending in 1–2 prescutellars (in both syntypes posterior half of the mesoscutum is obscured by pin; however, it is most probably that acrostichal setae lacking on prescutellar depression and, therefore, the number of acrostichal and dorsocentral setae is unusually small); 1 presutural intra-alar, 1 presutural supra-alar (no additional finer setae outside dorsocentrals); 3 notopleurals (1 seta on anterior part of notopleuron); 1 postsutural supra-alar and 1 rather strong seta on prealar area; 1 postalar; 2 long and 2 short scutellars; laterotergite with brown setae. **Legs**, including coxae, yellow; all femora shiny to subshiny anteriorly and posteriorly. Setae brown. Fore femur with short setae dorsally as well as inconspicuous anteroventral and posteroventral setae, whitish pilose ventrally. Fore tibia short setose. Mid and hind femora bare on anteroventral surface; with several short, spine-like posteroventral setae. Mid tibia short setose dorsally and ventrally; with 2 rows of very short spine-like setae ventrally. Hind tibia slender, short setose, setae only slightly stronger than usually, but much less than those on mid tibia; 1 very short seta in posteroapical comb. Basitarsus of all legs slender and short setose. **Wing** clear, pterostigma absent, veins depigmented; vein CuA+CuP absent in apical 2/3. Basal costal seta short to absent. Axillary excision slightly obtuse. Measurements: M_2_/dm = 1.6–1.7, M_4_ ap/mp = 1.6, wl/ww = 2.9. Halter yellow; calypter pale yellow with pale fringes. **Abdomen** brown, microtrichose, all setae brown. Chaetotaxy: posteromarginal setae on sides of tergites nearly as long as corresponding segment, discal setae shorter than marginals; dorsum of tergites with shorter setae. **Terminalia** as in Figure 14B (not dissected); epandrial lamella, cercus and “subcercal” process concolourous with abdominal tergites. Epandrial lamella rather subtriangular (lateral view), with elongated apical portion, bearing very long setae over lower margin, with short strong setae subapically. “Subcercal” process slender in comparison with rather broad cranial lobe of cercus. Phallus with gentle outgoing loop in middle.

**Female.** Unknown.

**Differential diagnosis:** *Rhamphomyia* (*P.*) *nudifemorata* sp. nov. can be easily distinguished from other Palaearctic species of the subgenus *Pararhamphomyia* by yellow legs, virtually bare femora on anteroventral side and dichoptic eyes. All other similar species (*R. hilariformis* Frey, *R. lividiventris* (Zetterstedt), Nearctic *R. flavirostris* Walker) have holoptic eyes and setose hind femur of legs. Female remains unknown.

**Distribution:** Russia (Amurskaya Province).

**Dates of occurrence:** July.

### 3.15. Rhamphomyia (Pararhamphomyia) plutenkoi sp. nov.

Zoobank link: urn:lsid:zoobank.org:act: 9AF0E8FB-4C3B-4CEA-B46A-7153464439AD

(Figure 15)

**Type material: HOLOTYPE, ♂**: Russia [Primorskiy Terr.], Artem [43°21′ N 132°11′ E], yellow pan traps, May 1996, leg. Plutenko (CZU).

**Diagnosis:** Small species of *Rhamphomyia* (*Pararhamphomyia*) (body 2 mm) with black setose body, biserial dorsocentrals and grey mesoscutum; postpedicel only 1.8× as long as broad, abdominal tergites 2–7 very light and silvery grey microtrichose.

**Etymology:** The species is named in honour of Andrej V. Plutenko, a well-known entomologist from Smolensk (Russia), the collector of the holotype.

**Figure 15:**
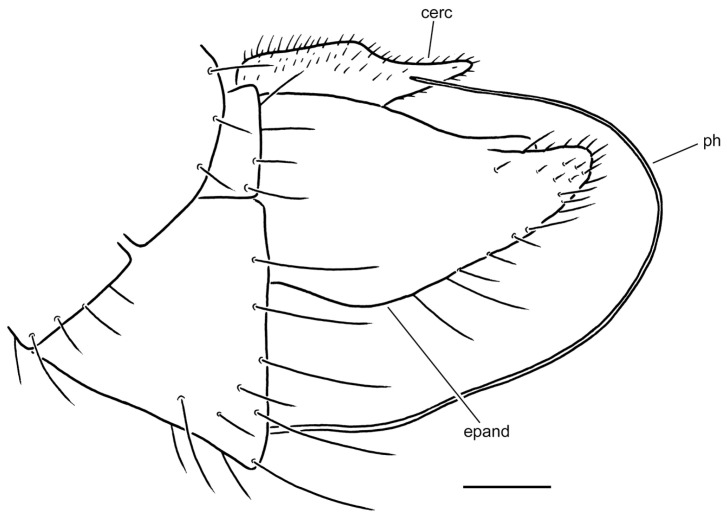
*Rhamphomyia* (*Pararhamphomyia*) *plutenkoi* sp. nov., male terminalia, lateral view, holotype. Abbreviations: cerc—cercus, epand—epandrium, ph—phallus. Scale bar 0.1 mm.

**Description: Male**. Length: body 2.0 mm (without genitalia), wing 2.2 mm. **Head** regions black, grey microtrichose. Eyes holoptic, upper ommatidia slightly enlarged. Frons bare. Ocellar setae black, half as long as frons, posterior part of ocellar triangle with single pair of additional setae. Face about 0.09 mm broad ventrally and 0.14 mm long, bare; clypeus microtrichose. Occiput rather sparsely, short, black setose, postocular row complete but distant from eye margin in middle part. Antenna black, scape and pedicel very short setose (longest setae about 0.05 mm long); antennomere ratio = 5:5:16:5 (postpedicel only 1.8× as long as broad). Labrum brown, shiny, only slightly shorter than head height. Palpus brown, short, bearing a few short setae. Gena narrow and microtrichose. **Thorax** black, light grey microtrichose, mesoscutum without distinct vittae. All setae black. Chaetotaxy: proepisternum with 1? seta on lower part and bare on upper part; prosternum bare; postpronotal lobe with 1 long and 3 shorter setae; about 14 rather broadly biserial acrostichals; 12–14 irregularly biserial dorsocentrals (moderately strong and slightly longer than acrostichals, 0.10 mm long in middle part of mesoscutum), ending in 2 longer prescutellars; 0–1 presutural intra-alar, 1 presutural supra-alar (with 2–3 additional shot setae outside of dorsocentrals in presutural area); 2–3 notopleurals (3–4 short fine setae on anterior part of notopleuron); 1–2 postsutural supra-alars and 3 setae in prealar area; 1 postalar; 2 long and 2 slightly shorter scutellars; laterotergite with several setae. **Legs** including coxae brown, microtrichose, black setose. Fore femur with complete rows of anteroventral and posteroventral setae (longest setae slightly longer than femur width), dorsal setae short. Fore tibia short setose, dorsal setation shorter than tibia width, no outstanding setae, ventral setae even shorter. Mid femur with complete rows of anteroventral and posteroventral setae slightly shorter than femur width. Mid tibia with 1 subbasal anterodorsal and 2–3 submedian posterodorsal long setae (nearly twice as long as tibia width); two almost regular rows of anteroventral and posteroventral setae (nearly as long as tibia width). Hind femur with row of strong anteroventral setae in apical third (nearly 1.5 times as long as femur width); similar row of slightly shorter posteroventral setae; dorsal setae short. Hind tibia with 2–3 setae dorsally (nearly 1.5 times as long as tibia width); anteroventral row of spinose setae (nearly as long as tibia width); posteroventral row consisting of shorter setae; 1 long seta in posteroapical comb. Basitarsus of all legs slender, short setose, with short ventral spine-like setae. **Wing** clear to slightly milky white, stigma brownish, radial veins yellowish brown, other veins depigmented; CuA+CuP absent in apical third. Basal costal seta present. Axillary excision slightly obtuse. Measurements: M_2_/dm = 1.2–1.3, M_4_ ap/mp = 1.6, wl/ww = 2.5. Halter yellow; calypter whitish yellow with dark fringes. **Abdomen** with sternites 1–7 brown, grey microtrichose, tergites 2–7 very light and silvery grey microtrichose, parts of segment 8 and parts of genital lamellae shiny to subshiny. Setae all dark. Chaetotaxy: posteromarginal setae on sides of tergites 2–4 slightly shorter than corresponding segments, on segments 5–6 1/3 as long as their corresponding segments, discal setae sparse and shorter than marginals; dorsum of tergites short setose. **Terminalia** as in Figure 15 (not dissected); phallus forming simple bow, slightly sinuated about base; cercus with shiny dorsal rim.

**Female.** Unknown.

**Differential diagnosis:** *Rhamphomyia* (*P.*) *plutenkoi* sp. nov. is closely allied to *R. curvula* Frey and *R. tibiella* Zetterstedt (and another species from the Nearctic Region) sharing with them (in addition to similar male genitalia) the following characters: dorsocentrals biserial, all body setae dark and mesoscutum grey microtrichose. The phallus in *R. tibiella* (and the Nearctic species) is S-shaped bent in middle and the epandrium bears a group of erected setae dorsally. The postpedicel is short in *R. pluntenkoi* sp. nov., whereas in *R. curvula* the postpedicel is at least 3× as long as broad. Moreover, a distinctive feature of the new species is the silvery abdomen. Female remains unknown.

**Distribution:** Russia (Primorskiy Terr.).

**Dates of occurrence:** May.

### 3.16. Rhamphomyia (Pararhamphomyia) sausai sp. nov.

Zoobank link: urn:lsid:zoobank.org:act: 9CE6936E-6465-4287-974C-2CAFC8FC119D

(Figure 16)

**Type material: HOLOTYPE** ♂, labelled: **CHINA**: Jilin prov. Da Gu Jia 20 km SEE of Jilin 43.793 N, 126.78 E, 350 m, 21.vi.2017 E.Jendek and O.Šauša leg. (CULSP). **PARATYPES:** same data as holotype (3 ♂, CULSP).

**Figure 16:**
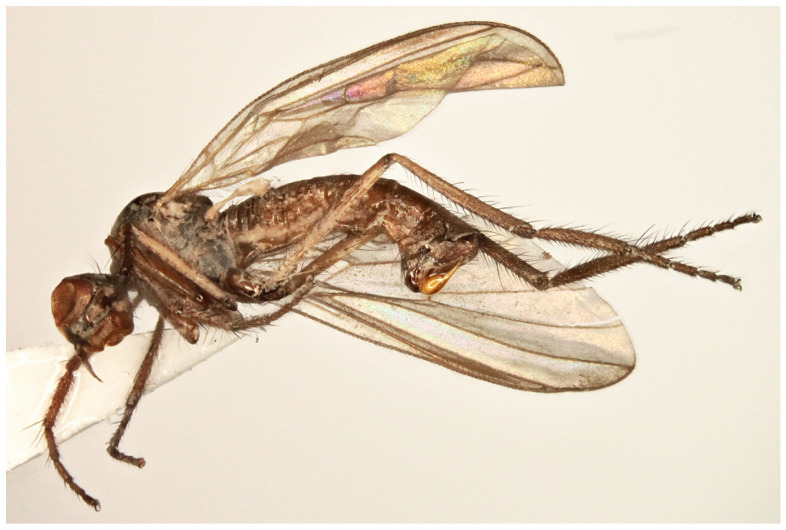
*Rhamphomyia* (*Pararhamphomyia*) *sausai* sp. nov., male habitus, lateral view.

**Diagnosis:** Medium-sized (body about 4 mm) black and black setose species of *Rhamphomyia* (*Pararhamphomyia*) with uniserial dorsocentrals and yellow halter; legs brown, mid leg with long setae, mid tibia straight; phallus swollen in basal part, cercus narrow apically without any projections.

**Etymology:** The species epithet, *sausai*, is a patronym in honour of Dr. Ondrej Šauša, collector of type series.

**Description: Male** (Figure 16). Length: body 3.5–4.1 mm, wing 3.5–3.9 mm. **Head** regions black, brownish grey microtrichose. Eyes holoptic, upper ommatidia much enlarged. Frons bare. Ocellar setae missing in all syntypes. Face about 0.18 mm long and 0.14 mm broad in middle, bare. Occiput sparsely black setose (setae on middle about 0.25 mm long, dorsally much shorter), postocular row incomplete. Antenna black, scape and pedicel with usual circlet of setae, postpedicel almost equally narrowing toward apex (stylus apex with short bare sensilla); antennomere ratio = 8–9:7–8:27–30:8. Labrum brown, shiny, shorter than head height. Palpus brown, very short, with only 2–4 short setae. Gena narrow, microtrichose; clypeus microtrichose. **Thorax** black, grey to brownish grey microtrichose. All setae black. Chaetotaxy: antepronotum with several fine and rather long setae; proepisternum with 3–4 fine setae in lower part and bare on upper part; prosternum bare; postpronotal lobe with 1 long seta and 3–4 additional much shorter setulae; acrostichals biserial, sparse (damaged in all specimens); dorsocentrals uniserial (damaged), ending in 1 very long prescutellar seta; presutural intra-alar small and fine, presutural supra-alar very long and strong; 2 strong notopleurals, sometimes 3 (lowermost much shorter), and additional, fine, small seta on anterior part of notopleuron; 1 long supra-alar and 1 long postalar; 2 long and 2 shorter scutellars; laterotergite with black setae. **Legs** light brown (femora paler than more distal parts), microtrichose and black setose: coxae concolorous with pleura. Fore femur very short setose, ventrally almost bare, only preapicals slightly longer. Fore tibia with short and fine, curled setae ventrally (shorter than tibia width); dorsal setae slightly longer, on apical third with 3 setae at least twice longer than tibia width, similar setae preapically and on posterodorsal side of fore basitarsus. Mid femur with short setae dorsally; anteroventral row sparse consisting of long setae (at least 2× longer than femur width), posteroventral row consisting of even longer setae (middle seta almost 0.45 mm long). Mid tibia with 2 long setae on basal part (more distal of them longer), 1 long preapical anterodorsal and 1 long seta ventrally in basal third of tibia (these 3 setae about 0.45 mm long, or half as long as tibia length). Hind femur with short setae dorsally, anteroventral row of long setae (longest setae in middle about 2× longer than femur width); posteroventral row of irregular, sparser and slightly shorter setae. Hind tibia ventrally with setae up to 2× longer than tibia width in basal third, shorter apically, several anterodorsal setae about 2× longer than tibia width over entire length; 1 short seta in posteroapical comb. Fore basitarsus with long setae posterodorsally (like those on fore tibia), similar setae forming preapical circlet, and with stronger setae ventrally as long as width of this tarsomere. Mid basitarsus short setose. Hind basitarsus ventrally short setose, dorsal setation slightly longer than basitarsus width, similar slightly elongate setae on second hind tarsomere. Remaining tarsal segments on all legs without conspicuous features. **Wing** clear, stigma distinct and darker, veins brown (yellow on basal part), CuA+CuP complete and well sclerotised throughout its length. Costal seta long, axillary excision 90º or slightly obtuse. Measurements: M_2_/dm = 1.6–1.7, M_4_ ap/mp = 2.4–2.5, wl/ww = 2.6–2.9. Halter yellow with dark base of stem; calypter brownish yellow with brown fringes. **Abdomen** brown (distinctly paler than thorax), sparsely microtrichose (appearing subshiny). Setae all black. Chaetotaxy: lateral posteromarginal setae shorter than length of segments (unusually long on dorsum of tergites); sternites short setose except longer setae on sternites 7 and 8. **Terminalia** with epandrial lamella and cercus brownish, microtrichose, black setose; hypandrium yellowish brown, shiny, bare; phallus yellow. Epandrial lamella elongate with upturned tip; bearing long fine setae over lower margin. Hypandrium very narrow, almost strip-like in ventral view. Cercus broader on basal part, narrower and almost parallel-sided on apical part (lateral view). Phallus gently curved (almost straight apically), swollen on basal part.

**Female.** Unknown.

**Differential diagnosis:** The species described above belongs to the *R.* (*Pararhamphomyia*) *ciliatopoda* group (as specified by Saigusa [24] and was further revised by Barták and Kubík [3]. *Rhamphomyia sausai* sp. nov. is most similar to *R*. *setulosa* Saigusa described from Honshu [25] and also known to us from the Korean Peninsula and the south of Russian Far East (Barták & Shamshev, unpublished data). The new species can be distinguished from *R*. *setulosa* primarily in chaetotaxy of fore and mid legs. In *R*. *sausai* sp. nov., fore basitarsus is very long setose dorsally and mid tibia bears very long ventral and dorsal setae. In *R*. *setulosa,* fore basitarsus short setose and mid tibia lacks long ventral and dorsal setae. In addition, there some differences between these species in shape of cercus and epandrial lamella. In *R*. *sausai* sp. nov., the cercus and epandrial lamella are much narrower apically than in *R*. *setulosa*. Also, *R*. *sausai* sp. nov. could be compared with *R. curvitibia* Saigusa [26]. However, in the new species the cercus has no ventral process and the mid tibia is straight. The male terminalia of *R. sausai* sp. nov. are somewhat similar to *R. maai* Saigusa [25], but the phallus of the new species is strongly swollen basally, and the latter species has yellow coxae and much shorter setose fore tibiae and tarsi. Females of species from this group of specie are mostly unknown, except *R. rotundicauda* Saigusa and *R. thaiciliata* Barták & Kubík [3,25], and they have pennate legs. However, in the female of *R*. *tibialis,* the legs are not pennate, the wing is broadened and brown (Barták & Shamshev, unpublished data). In spite of this, the description above is based largely on combination of characters present at least in one of the four of rather damaged males we had at our disposal, we do not hesitate to describe it as a new and well-characterised species.

**Distribution:** China (Jilin).

### 3.17. Rhamphomyia (Pararhamphomyia) schachti sp. nov.

Zoobank link: urn:lsid:zoobank.org:act: 452DFAF4-F18A-424E-B698-CC251C3670B2

(Figure 17A,B)

**Type material: HOLOTYPE ♂**, labelled: Spain, Pr. Granada, Sierra Nevada, Puerto d.l. Ragua [37°06′ N 3°01′ E], 1700 m, 13.vii.1979, leg. W. Schacht (CZU). **PARATYPE: SPAIN**: same data as holotype (1 ♀, CZU).

**Figure 17:**
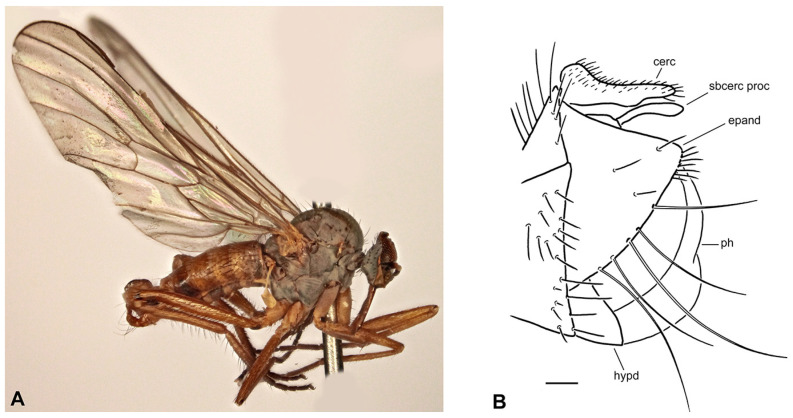
*Rhamphomyia* (*Pararhamphomyia*) *schachti* sp. nov., male holotype. (**A**) = Habitus, lateral view. (**B**) = Terminalia, lateral view. Abbreviations: cerc—cercus, epand—epandrial lamella, hypd—hypandrium, ph—phallus, sbcerc proc—subcercal process. Scale bar 0.1 mm.

**Diagnosis:** Medium-sized species (body about 5 mm) of *Rhamphomyia* (*Pararhamphomyia*) with uniserial dorsocentral setae, yellow legs and dark palpus; antenna with scape and pedicel yellow, mesoscutum light grey with distinct brown vittae down rows of acrostichal and dorsocentral setae. Male: eyes holoptic; hind legs deformed near the “knee”. Female: eyes dichoptic, legs pennate.

**Etymology:** The species is named in honour of the collector of types.

**Description: Male** (Figure 17A). Length: body 4.9 mm (without genitalia), wing 6.4 mm. **Head** regions black, light grey microtrichose. Eyes holoptic, upper ommatidia enlarged. Frons, bare. Ocellar setae black, very short and fine (0.11 mm long, shorter than distance between outer margins of dorsal ocelli), ocellar triangle with 1 pair of additional slightly shorter setae. Face about 0.21 mm broad ventrally and 0.20? mm long, bare; clypeus microtrichose. Occiput rather sparsely, short, black setose, setae rather strong (especially those forming hind row), arranged in almost two rows, postocular row almost complete but irregular in middle part. Antenna with scape and pedicel yellow, short setose (longest setae about 0.08 mm long), postpedicel and stylus black; antennomere ratio = 7:9:34:12. Labrum yellowish brown, shiny, about as long as head height. Palpus brown, short, bearing a few short setae. Gena narrow, shiny in hind part. **Thorax** black, light grey microtrichose, mesoscutum with 3 brown vittae under rows of acrostichal and dorsocentral setae. All setae black. Chaetotaxy: proepisternum with 1 seta on lower part and bare on upper part; prosternum bare; postpronotal lobe with 1 strong and 4–6 much shorter setae; about 12? narrowly biserial acrostichals very short (0.07 mm long—half as long as distance between rows of acrostichals and dorsocentrals); about 10 uniserial dorsocentrals longer than acrostichals (nearly 0.15 mm in middle part of row), ending in 1–2 long prescutellars; 1 strong presutural intra-alar; 1 strong presutural supra-alar (with 1–3 additional setae between presutural intra-alar and supra-alar setae); 2 notopleurals (1–2 short fine setae on anterior part of notopleuron); 1–2 postsutural supra-alars (with 0–1 seta in prealar area); 1 postalar; 2 long and 2 shorter (half as long) scutellars; laterotergite with several setae. **Legs**, including coxae, yellow (fore coxa and tarsus slightly darkened), microtrichose, black setose. Fore femur short setose, anteroventral and posteroventral setae much shorter than femur width. Fore tibia short setose, bearing contrastingly strong and somewhat flattened anterior (ad?) preapical seta. Mid femur short setose, anteroventral and posteroventral setae rather spinose but short. Mid tibia short setose, anteroventral and posteroventral setae rather spinose and short. Hind femur with anteroventral incision and black posteroventral protuberance preapically; covered with short setae (longest setae nearly as long as femur width: anteroventrals in apical third and several posteroventrals in basal half). Hind tibia with subbasal black protuberance opposite of protuberance on hind femur; dorsal setae slightly longer than tibia width, ventral setae short; 1 long seta in posteroapical comb. Fore basitarsus slender, short setose dorsally, ventrally with several very fine setae longer than diameter of this tarsomere, also tarsomeres 2 and 3 with similar setae ventrally; mid basitarsus slender, short setose; hind basitarsus very slightly swollen, dorsal setae almost twice as long as diameter of this tarsomere. **Wing** clear, stigma slightly darker, veins yellowish brown; vein CuA+CuP almost complete but indistinct in middle. Basal costal seta present. Axillary excision 90º. Measurements: M_2_/dm = 1.4, M_4_ ap/mp = 1.9, wl/ww = 3.1. Halter yellow; calypter yellow with dark fringes. **Abdomen** extensively yellow, blackish only near extreme base, almost entirely microtrichose, only parts of tergites 5–6 shiny. Setae all dark. Chaetotaxy: posteromarginal setae on sides of tergites 2–4 nearly as long as corresponding segment, shorter on remaining segments; discal setae slightly shorter than marginals; dorsum of tergites shorter setose. Tergite 5 with narrow finger-like projection at ventralmost corner. **Terminalia** as in Figure 17B (not dissected); epandrial lamella and cercus almost concolourous with abdominal tergites, dark setose, epandrial lamella microtrichose, cercus with black dorsal rim, shiny. Epandrial lamella subtriangular (lateral view), with several very long curved setae ventrally and with cluster of setae apically. Cercus and “subcercal” process narrow.

**Female.** Length: body 5.7 mm, wing 5.6 mm. Similar to male except as follows. Eyes broadly dichoptic, all facets of subequal size. Frons 0.20 mm broad in middle and 0.21 mm long, with 2–3 rather strong and long setae on each side. Antennomere ratio = 6:8:30:11. Ocellar setae strong, longer than frons. Postocular row of setae interrupted in middle. Fore femur and tibia as in male, also preapical anterior seta on tibia present. Mid femur short setose, posteroventral setae shorter than femur width and slightly flattened in apical third. Mid tibia short setose, anterodorsal and posteroventral setae form rather dense rows but they seem to be not flattened. Hind femur and tibia unmodified. Hind femur with anteroventral row of setae half as long as femur width, posteroventrals slightly longer and distinctly flattened in apical third. Hind tibia broadly pennate on both sides. Basitarsi of fore and mid legs as in male, hind basitarsus slender, broadly pennate dorsally. Wing as in male; measurements: M2/dm = 1.3–1.4, M_4_ ap/mp = 1.4–1.5, wl/ww = 3.2. Abdomen yellow, darkened about base and lateral parts of basal tergites, entirely microtrichose. Posteromarginal setae on sides of tergites 2–4 slightly shorter than their segments, on segment 5 they are half as long as the segment, and on remaining segments they are shorter; discal setae slightly shorter than marginals.

**Differential diagnosis:** *Rhamphomyia* (*Pararhamphomyia*) *schachti* sp. nov. belongs to the species group of *Pararhamphomyia* with uniserial dorsocentral setae, yellow legs and dark palpus. The new species differs from other species of this group (except *R. barbata* (Macquart)) in having the two basal antennomeres yellow and light grey mesoscutum with distinct brown vittae down rows of acrostichal and dorsocentral setae. Moreover, the male hind legs are deformed near the “knee”, and the female legs are pennate. Only two Palaearctic species share these characters: *R. schachti* sp. nov. and *R. barbata*. However, the male of the latter species has cercus broad and bilobate, and the hind femur bears a cluster of posteroventral setae near the tip (about as long as femur width). The female of *R. barbata* has mid tibia pennate and hind basitarsus not pennate.

**Distribution:** Spain.

**Dates of occurrence:** July.

### 3.18. Rhamphomyia (Pararhamphomyia) seticauda sp. nov.

Zoobank link: urn:lsid:zoobank.org:act: 4140EAE6-B64F-4D29-905F-5E1DC4D2B549

(Figure 18A–C)

**Type material: HOLOTYPE ♂**, labelled: [in Cyrillic; **RUSSIA**, Primorskiy Territory] Yuzh. [=Yuzhnoe] Primorje/Kamenushka [43°37′ N 132°13′ E], 14.vi.1984/A. Shatalkin (ZMMU). **PARATYPE: RUSSIA. Primorskiy Ter.:** same locality as holotype, 13.vi.1984, A. Shatalkin (1 ♂, CULSP).

**Figure 18:**
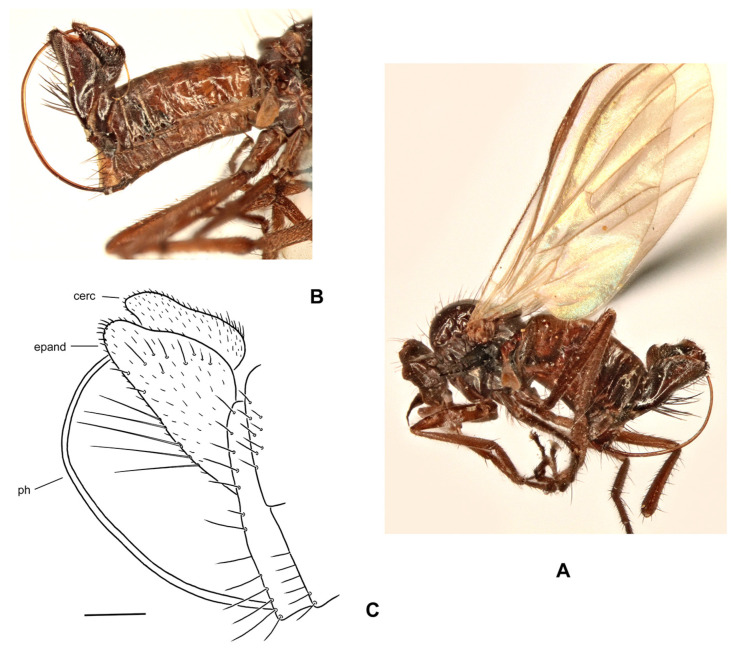
*Rhamphomyia* (*Pararhamphomyia*) *seticauda* sp. nov., male holotype. (**A**) = Habitus, lateral view. (**B**) = Abdomen, lateral view. (**C**) = Terminalia, lateral view. Abbreviations: cerc—cercus, epand—epandrial lamella, ph—phallus. Scale bar 0.1 mm.

**Diagnosis:** Small species (body about 2.5 mm) of *Rhamphomyia* (*Pararhamphomyia*) with subshiny mesoscutum, uniserial dorsocentral setae, dark legs, yellow halters, entirely black setose body, hind basitarsus slightly swollen, incomplete vein CuA+CuP and unmodified male pregenital segments.

**Etymology:** The species is named after the peculiar strong setae on the epandrium.

**Description: Male** (Figure 18A). Length: body 2.2? mm (without genitalia), wing 3.0 mm. **Head** regions brownish black, grey microtrichose. Eyes holoptic, upper ommatidia enlarged. Frons represented by small subtriangular spaces below ocellar triangle and above antennae, bare. Ocellar setae black, half as long as frons. Face about 0.10? mm broad ventrally and 0.13 mm long, bare. Occiput rather sparsely, short, black setose. Antenna black, scape and pedicel rather short setose; antennomere ratio = 6:5:22:10. Labrum brown, shiny, 2/3 as long as head is high. Palpus brown, short, bearing only two setae. Gena narrow, microtrichose; clypeus microtrichose. **Thorax** brownish black; mesoscutum subshiny, without vittae (only faintly covered with microtrichiae); pleurae microtrichose. All setae black. Chaetotaxy: proepisternum with 2–3 setae on lower part and bare on upper part; prosternum bare; postpronotal lobe with 1 strong, long and 2–3 much shorter setae; about 20 biserial, fine acrostichals (0.10 mm long); 5–6 uniserial dorsocentrals slightly longer than acrostichal setae, ending in 2 strong prescutellars; 1 fine presutural intra-alar similar to dorsocentrals, 1 strong presutural supra-alar (no other setae outside dorsocentrals); 3–4 notopleurals (anterior part of notopleuron bare); 1 supra-alar; 1 postalar; 2 long and 2 much shorter scutellars; laterotergite with black setae. **Legs** including coxae brown, microtrichose, black setose. Fore femur with short setae, anteroventrals and posteroventrals very fine, nearly half as long as femur width. Fore tibia with short and fine uniform setation dorsally about as long as tibia width (some of them rather upright standing, setae not differentiated except preapicals). Mid femur without anteroventrals in basal half, bearing 6 fine anteroventral setae in apical half (nearly as long as femur width); posteroventrals present throughout, and they are slightly longer than anteroventrals. Mid tibia with 2 anterodorsal (1 on basal third and 1 preapically) and 2 posterodorsal (1 on apical third and 1 preapically) long setae, otherwise short setose. Hind femur with row of fine anteroventral setae (nearly as long as femur width); posteroventrals absent; 1 long anterodorsal seta in apical third. Hind tibia slender; with 0–1 anterodorsal and 5–6 posterodorsal setae (slightly longer than tibia width); ventral setae short; 1 very short seta in comb at tip. Basitarsus of fore and mid legs slender and short setose; hind basitarsus slightly swollen, short setose. **Wing** clear, stigma slightly darker, veins yellowish-brown; CuA+CuP incomplete, absent in apical 2/3. Costal seta present. Axillary excision slightly obtuse. Measurements: M_2_/dm = 1.3, M_4_ ap/mp = 1.4, wl/ww = 2.6. Halter yellow; calypter pale brown with dark fringes. **Abdomen** brown, microtrichose, only extreme base of epandrium and small part of sternite 8 shiny. Setae all dark. Chaetotaxy: posteromarginal setae on sides of tergites 2–3 slightly shorter than corresponding segment, remaining segments shorter setose, discal setae subequal to marginals. Dorsum of tergites with shorter setae. **Terminalia** (Figure 18B,C, not dissected) large in comparison with short abdomen; epandrial lamella and cercus concolourous with abdominal tergites, black setose, phallus yellowish brown. Epandrial lamella subtriangular (lateral view); with strong, long setae ventrally and comb of short setae on apex (best visible from behind, setae about 0.10 mm long). Cercus similar to *R. subcurvitibia* sp. nov.; “subcercal” process obviously absent.

**Female.** Unknown.

**Differential diagnosis:** *Rhamphomyia* (*P.*) *seticauda* sp. nov. belongs to the species-rich group of *Pararhamphomyia* with uniserial dorsocentral setae, dark legs, yellow halters and entirely black setose body. Certain combination of characters (pregenital segments not modified, hind legs simple, mesoscutum subshiny, phallus simply bowed and incomplete vein CuA+CuP) this new species shares (inside the Palaearctic fauna) only with *R. fuscapicis* Saigusa [25]. However, the latter species has short epandrium bearing ordinary setae, wing distinctly bicolorous (infuscated apically), mid tibia with very long anterodorsal seta before middle and hind basitarsus long setose dorsally. The new species seems to be closely allied to *R. subcurvitibia* sp. nov., which has dark halter and finer setae on the epandrium. Additional allied species are members of the *R. takagii* group, with similarly faintly microtrichose mesoscutum and rather bare thorax. However, all known species of this group have modified pregenital segments in the male and/or pale legs.

**Distribution:** Russia (Primorskiy Territory).

**Dates of occurrence:** June.

### 3.19. Rhamphomyia (Pararhamphomyia) spinicauda sp. nov.

Zoobank link: urn:lsid:zoobank.org:act: 276D8F10-5446-4787-9BAD-FE4BA5B4B23B

(Figure 19A,B)

**Type material: HOLOTYPE** ♂, labelled: [in Cyrillic, **RUSSIA**] Amursk. obl. [=Amurskaya oblast, province], g. [=gorod, tawn] Zeya [53°44′ N 127°15′ E], 13.vii.1978/A. Shatalkin (ZMMU). **PARATYPES: RUSSIA. Amurskaya Prov.:** same data as holotype (7 ♂, ZMMU; 6 ♂, ZISP); same locality as holotype: 24.vi.1979, leg. A. Shatalkin (1 ♂, CULSP); 21.vii.1981, leg. O. Gorbunov (1 ♂, ZMMU); 29.vii.1981, leg. A. Shatalkin (1 ♀, CULSP); 30.vii.1978, A. Shatalkin (1 ♂, ZISP; 2 ♂ ZMMU); 3.vii.1978, leg. A. Shatalkin (5 ♂, ZMMU); 6.vii.1981, leg. A. Ozerov (2 ♂, ZMMU); 8.vii.1978, A. Shatalkin (1 ♂, ZISP); 9.vii.1978, A. Shatalkin (1 ♂, ZISP); 34 km N Zeya, 5.vii.1982, Zlobin (4 ♂, ZISP); 52 km N Zeya, 1.vii.1982, Zlobin (2 ♂, ZISP).

**Figure 19:**
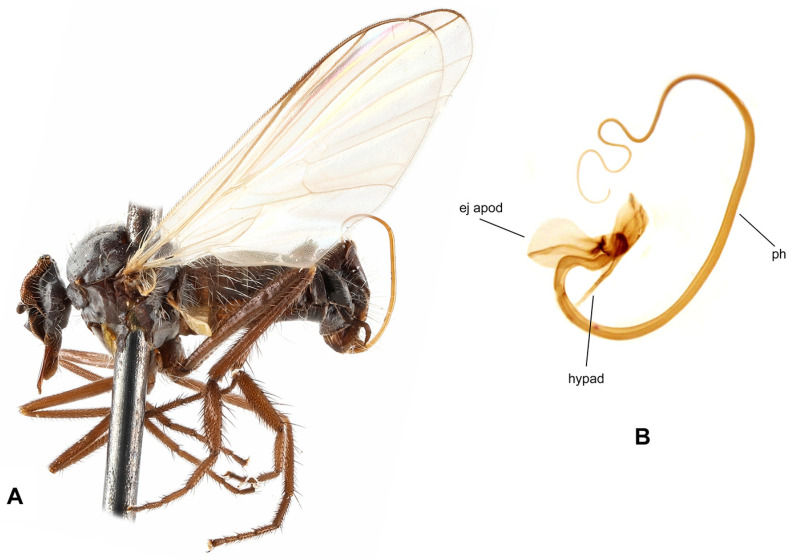
*Rhamphomyia* (*Pararhamphomyia*) *spinicauda* sp. nov., male paratype. (**A**) = Habitus, lateral view. (**B**) = Hypandrium and phallus, lateral view. Abbreviations: ej apod—ejaculatory apodeme, hypd—hypandrium, ph—phallus.

**Diagnosis:** Relatively small species (body 3–4 mm) of *Rhamphomyia* (*Pararhamphomyia*) with light grey microtrichose mesoscutum, uniserial dorsocentral setae, incomplete vein CuA+CuP and white setose body. Male: hind basitarsus slightly swollen; phallus forms large and simple bow, twice S-shaped bent before apex. Female: wing yellowish.

**Etymology:** The species is named after the sharp tooth at the end of male abdomen.

**Description: Male** (Figure 19A). Length: body 3.0–3.9 mm (without genitalia), wing 3.0–3.7 mm. **Head** regions black to brownish black, light grey microtrichose. Eyes holoptic, upper ommatidia enlarged. Frons bare. Ocellar setae white, 1/3 as long as frons, ocellar triangle with 3 pairs of additional short setae. Face microtrichose, about 0.14 mm broad ventrally and 0.18 mm long, bare. Occiput rather sparsely, moderately long, white setose; postocular row complete but distant from eye margin in middle. Antenna with scape and pedicel brown, postpedicel and stylus black, scape and pedicel short setose; antennomere ratio = 5:7:22:13. Labrum brown, shiny, 2/3 as long as head height. Palpus brown, short, bearing a few setae. Gena narrow, microtrichose; clypeus microtrichose. **Thorax** black, light grey microtrichose, mesoscutum without distinct vittae. All setae white. Chaetotaxy: proepisternum with 3–4 setae on lower part and bare on upper part; prosternum bare; about 8–14 narrowly biserial acrostichals; 6–10 uniserial dorsocentrals (but sometimes with 1 seta standing outside row in postsutural part) slightly longer than acrostichals (0.30 mm in middle of the row), ending in 2–3 prescutellars; 1 presutural intra-alar (plus 0–1 seta); 1 presutural supra-alar (usually no additional setae); 1 long postpronotal and several shorter setae; 3 notopleurals, (1–4 setae on anterior part of notopleuron); 1 supra-alar; 1 postalar; 2 long and 2 shorter (1/2–2/3 as long) scutellars; laterotergite with numerous white setae. **Legs** brown, microtrichose, white setose proximally and brown setose distally. Coxae brownish black, microtrichose, pale setose. Fore femur with rows of anteroventral and posteroventral very short setae, only just before tip they nearly half as long as femur width. Fore tibia with almost uniform short setation (nearly as long as tibia width), setae not differentiated except preapicals. Mid femur with a few longer setae ventrally near base; anteroventral row irregular, setae about as long as femur width in apical part, shorter in middle; posteroventrals longer than anteroventrals and more regular. Mid tibia with 1–3 anterodorsal short setae (nearly as long as tibia width) and 3–4 longer posterodorsal setae (nearly 1.5 times as long, only preapicals somewhat longer), short setose ventrally. Hind femur with anteroventral setae longer in about basal half (as long as or slightly longer than femur width) and short in apical half, posteroventrals slightly shorter than anteroventrals. Hind tibia with 2–4 anterodorsals and 4–6 posterodorsals nearly 1.5 times as long as tibia width, ventral setae short; 1 long seta in posteroapical comb. Fore and mid basitarsi slender, short setose; hind basitarsus slightly swollen, with several dorsal setae as long as setae on hind tibia. **Wing** clear, stigma yellowish, veins pale yellowish; vein CuA+CuP depigmented throughout. Costal seta present, axillary excision 90º. Measurements: M_2_/dm = 1.4–1.6, M_4_ ap/mp = 1.6–1.8, wl/ww = 2.5–2.6. Halter yellow, calypter yellow with white fringes. **Abdomen** black, light grey microtrichose (bases of tergite 7 and syntergosternite 8 shiny, also extreme base of epandrium shiny). Setae all white. Chaetotaxy: posteromarginal setae on sides of tergites 2–6 slightly shorter than corresponding segments, discal setae subequal to marginals; dorsum of tergites shorter setose. Segment 7 with long and sharp lateral tooth, tergite and sternite 8 fused into syntergosternite. **Terminalia** as Figure 19B; epandrial lamella and cercus concolourous with abdominal tergites; epandrial lamella with numerous long white setae over lower margin; cercus covered with dark setulae. Epandrial lamella subtriangular. Hypandrium divided into two lateral sclerites, membranous ventrally. Cercus simple (“subcercal” process absent). Phallus thin, broadly bowed, twice S-shaped bent before apex.

**Female.** Length of body and wing 3.2 mm. Similar to male but with the following exceptions. Eyes broadly dichoptic, dorsalmost facets smaller than ventral ones. Frons 0.14? mm broad and 0.18 mm long, with a few short setae on each side. Labrum slightly shorter than head is high. Thorax as in male, only setae slightly shorter (dorsocentrals about 0.15 mm long). Legs similarly coloured as in male but differently setose. Fore femur short setose, ventrally almost bare (except on base). Fore tibia short setose. Mid femur with irregular anteroventral and posteroventral setation nearly as long as femur width. Mid tibia with 1–2 anteroventral and posterodorsal setae scarcely differentiated and about as long as tibia width. Hind femur with row of anteroventral setae 1/3 (in middle) to 1/2 (near apex) as long as femur is high, denser posteroventral setae equally long. Hind tibia thin, with several posterodorsal setae nearly as long as tibia width, ventral setae short. Basitarsi of all legs slender, short setose, with short ventral spines. Mid basitarsus slightly bent ventrally. Wing yellowish, stigma indistinct; measurements: M_2_/dm = 1.5, M_4_ ap/mp = 1.5, wl/ww = 3.0. Abdomen brown, rather thinly grey microtrichose, sternite 8 with shiny longitudinal stripe. Posteromarginal setae on tergites 1–3 half as long as their corresponding segment, shorter on remaining tergites, discal setae shorter than marginals.

**Differential diagnosis:** *Rhamphomyia* (*P.*) *spinicauda* sp. nov. is, alongside *R*. (*P*.) *haladai* sp. nov., the only known Palaearctic *Pararhamphomyia* with uniserial dorsocentrals and white setose mesoscutum. Another similar species from Uzbekistan (Mt. Chimgan), still undescribed, also with pale uniserial dorsocentrals, has the process on the segment 7 broadly ovate. However, the colour of the setae may vary [27]. The most allied species are *R. tienshanensis* Barták and *R. subsultans* Frey; however, both have at least biserial dorsocentral setae [22,28]. The phallus is similarly shaped as in *R. mendicula* Frey; however, this species has setose prosternum [29].

**Distribution:** Russia (Amurskaya Province).

**Dates of occurrence:** June–July.

### 3.20. Rhamphomyia (Pararhamphomyia) spiraliseta sp. nov.

Zoobank link: urn:lsid:zoobank.org:act: 2CDE5917-259A-45B4-8203-CABF53FDAB1A

(Figure 20A,B)

**Type material: HOLOTYPE ♂**, labelled: [in Cyrillic, **RUSSIA**, Yakutia], r. [=reka, river] Indigirka/ust. r. [=ustje reki, mouth of river] Injali [65°14′ N 143°08′ E]/23.vi.1976, V. Kovalev//ostepnennyj sklon rechnoj terrasy [=steppe slope of a river terrace] (ZMMU). **PARATYPES: RUSSIA. Yakutia:** Indigirka River, Momskij District, 28.vi.1986, V. Kovalev (1 ♂, PCMB); same locality, 2.vii.1976, V. Kovalev (1 ♂, PCMB).

**Diagnosis:** Small species (wing about 3 mm) of *Rhamphomyia* (*Pararhamphomyia*) with black setose body, biserial dorsocentral setae, wing clear to slightly milky white on base, halter brown, mid tibia with distorted ventral rows of setae, mid basitarsus with spine-like setae.

**Etymology:** The species is named after the peculiarly spirally distorted ventral row of setae on mid tibia.

**Figure 20:**
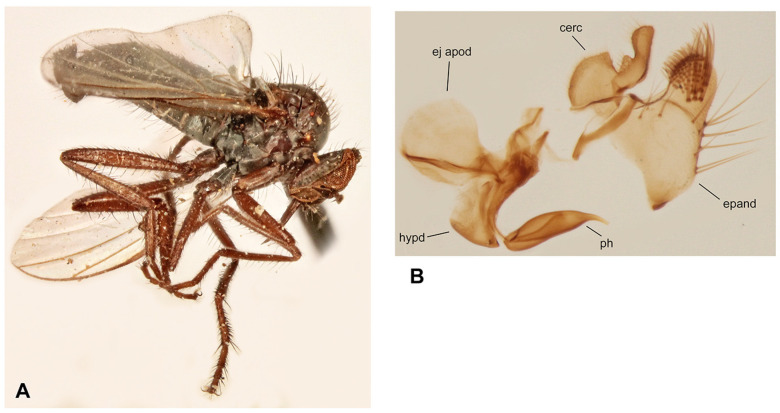
*Rhamphomyia* (*Pararhamphomyia*) *spiraliseta* sp. nov., male. (**A**) = Habitus, paratype, lateral view. (**B**) = Terminalia, holotype, lateral view. Abbreviations: cerc—cercus, epand—epandrial lamella, ej apod—ejaculatory apodeme, hypd—hypandrium, ph—phallus.

**Description: Male** (Figure 20A). Length: body 2.9–3.2 mm (without genitalia), wing 3.0–3.2 mm. **Head** regions black to brownish black, greyish microtrichose. Eyes holoptic, upper ommatidia enlarged. Frons bare. Ocellar setae black, moderately strong, half as long as frons, ocellar triangle without additional setae. Face 0.15 mm broad ventrally and 0.10 mm long, bare. Occiput sparsely black setose, postocular row complete but distant from eye margin in ventral 2/3. Antenna with scape and pedicel brown, short setose (longest setae 0.07 mm long), postpedicel and stylus black; antennomere ratio = 6:6:21:9. Labrum brown, shiny, 3/4 as long as head height. Palpus brown, bearing a few rather long setae (longest preapicals 0.20 mm long). Gena narrow, mostly microtrichose; clypeus microtrichose. **Thorax** brownish black, brownish grey microtrichose, mesoscutum appearing light in anterior view but getting dark in posterior view, without vittae. All setae black. Chaetotaxy: proepisternum with 5–6 long setae on lower part and bare on upper part; prosternum bare; 15–18 biserial acrostichals; 11–14 irregularly biserial dorsocentrals, ending in 2 prescutellars; both acrostichals and dorsocentrals moderately strong and about 0.15–0.20 mm long in middle of rows; 1 presutural intra-alar; 1 presutural supra-alar (plus 2–4 rather long setae outside of dorsocentrals in presutural area); 1 long postpronotal and 5–6 slightly shorter setae; 3 notopleurals, (with additional 2–4 fine, rather long setae on anterior part of notopleuron); 2 postsutural supra-alars (4–6 setae on prealar area in transverse row reaching dorsocentrals); 1 long and 1 short postalars; 4 scutellars (lateral pair half as long as apical pair); laterotergite with numerous setae. **Legs** including coxae brown, microtrichose, black setose. Fore femur with rows of anteroventral and posteroventral, moderately long, sparse setae (as long as femur width), dorsal setae short. Fore tibia with posterodorsal setae in basal third twice as long as tibia width and shorter apically, ventral setae short. Mid femur with row of anteroventral, sparse, short setae (shorter than femur width) and similar row of posteroventrals slightly longer than femur width. Mid tibia with equally spread 4 strong setae dorsally (nearly 0.30 mm long; basal and preapical setae in anterodorsal position and both middle setae in posterodorsal position); ventral rows peculiar: anteroventral row restricted to basal 1/4 of tibia and row, which starts proximally in posteroventral position is spirally distorted to ventral side distally (these spinose setae are longest in basal part of posteroventral row, where they are slightly longer than tibia width). Hind femur with anteroventral row of long setae in apical third (nearly twice as long as femur width), posteroventral setae shorter than anteroventrals except 1 posteroventral subapical seta; dorsal setae short. Hind tibia with 6–8 irregularly arranged setae dorsally (about 1.5 times as long as tibia width) and with anteroventral row of spinose setae similar to posteroventral setae on mid tibia; 1 long seta in posteroapical comb. Fore basitarsus slender, short setose; mid basitarsus slender, covered with conspicuous spinose setae slightly longer than its diameter; hind basitarsus rather slender, short setose. **Wing** clear to slightly milky white on base, stigma hyaline, veins yellowish or hyaline, CuA+CuP absent in apical third. Costal seta present, axillary excision very slightly acute. Measurements: M_2_/dm = 1.2–1.5, M_4_ ap/mp = 1.8, wl/ww = 2.7. Halter brown, calypter brownish with dark fringes. **Abdomen** brown, almost light grey microtrichose in lateral view and dark brown in dorsal view. Chaetotaxy: posteromarginal setae on sides of tergites 2–3 subequally long as corresponding segment, shorter on remaining segments, discal setae slightly shorter than marginals; dorsum of tergites short setose. **Terminalia** as in Figure 20B, concolourous with abdominal tergites. Epandrial lamella with preapical tuft of setae directed upwards. Hypandrium shiny, exposed and firmly attached to basal swollen part of phallus (as usually in species of *R. tranversipyga* group). Cercus with small subtriangular projection. Phallus with extremely short apical slender part in comparison with long swollen part.

**Female.** Unknown (see discussion under *R. indigirka* sp. nov.).

**Differential diagnosis:** *Rhamphomyia* (*Pararhamphomyia*) *spiraliseta* sp. nov. belongs to the *R*. (*P*.) *pusilla* group of species [3]. *Rhamphomyia* (*P.*) *spiraliseta* sp. nov. differs from all Palaearctic species of the *R. pusilla* complex in having distorted ventral rows of setae on mid tibia and spinose mid basal tarsomeres. At least three allied species occur in the Nearctic region, which have similarly distorted rows of ventral setae on the mid tibia, spinose mid basal tarsomeres and similar male genitalia. However, they have deformed legs and slightly different genitalia. For probable female characters, see the discussion under *R. indigirka* sp. nov.

**Distribution:** Russia (Yakutia).

**Dates of occurrence:** June–July.

### 3.21. Rhamphomyia (Pararhamphomyia) subcurvitibia sp. nov.

Zoobank link: urn:lsid:zoobank.org:act: 8B7FE807-5D2A-4AC2-A61E-DDE22273B558

(Figure 21A–D)

**Type material: HOLOTYPE ♂**, labelled: [in Cyrillic; **RUSSIA**, Primorskiy Territory] Yuzh. [=Yuzhnoe] Primorje/Kamenushka [43°37′ N 132°13′ E], 21.vi.1984/A. Shatalkin (ZMMU). **PARATYPES: RUSSIA, Primorskiy Ter.:** on one pin with holotype [in copula] (1 ♀, ZMMU); same locality, 16.vi.1984 (3 ♀, ZMMU and CULSP).

**Diagnosis:** Small species of *Rhamphomyia* (*Pararhamphomyia*) with subshiny mesoscutum, uniserial dorsocentral setae, dark legs, entirely black setose body and dark halter. Male: hind tibia strongly curved. Female: hind femora with flattened setae, hind tibia slightly broadened near base, wing dark brown.

**Etymology:** The species is named after the peculiarly curved tibia (specific name “curvitibia” is preoccupied).

**Figure 21:**
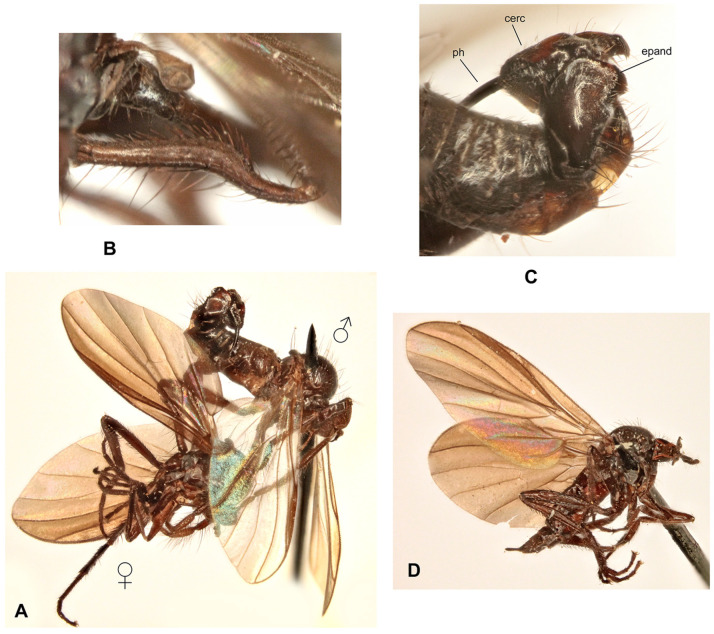
*Rhamphomyia* (*Pararhamphomyia*) *subcurvitibia* sp. nov. (**A**) = Male (holotype) and female in copula. (**B**) = Male hind tibia, anterior view. (**C**) = Male postabdomen, lateral view. (**D**) = Female habitus, lateral view. Abbreviations: cerc—cercus, epand—epandrial lamella, ph—phallus.

**Description: Male** (Figure 21A). Length: body 2.9? mm (without genitalia), wing 3.1 mm. **Head** regions brownish black to brown, grey microtrichose. Eyes holoptic, upper ommatidia enlarged. Frons bare. Ocellar setae black, half as long as frons, ocellar triangle with 2 pairs of additional short setae. Face about 0.09 mm broad ventrally and 0.14 mm long, bare. Occiput rather sparsely, short, black setose. Antenna black; scape and pedicel slightly paler than postpedicel, rather short setose (longest setae about 0.06 mm long); antennomere ratio = 6:6:25:9. Labrum brown, shiny, half as long as head height. Palpus brown, short, bearing a few short setae and 1 longer preapical seta. Gena narrow, microtrichose; clypeus microtrichose. **Thorax** brownish black, black setose; mesoscutum subshiny, without vittae (only faintly microtrichose), pleurae microtrichose. Chaetotaxy: proepisternum with 2–3 setae in lower part; prosternum and upper part of proepisternum bare; postpronotal lobe with 1 strong, long and 2–3 much shorter setae; acrostichals 1–2-serial (about 12 setae), fine; 5–6 uniserial dorsocentrals slightly longer than acrostichals (0.15 mm), 3 strong prescutellars; 1 presutural intra-alar seta (somewhat longer than dorsocentrals), 1 strong presutural supra-alar seta (with 1 additional short seta); 3 notopleurals (1 seta on anterior part of notopleuron); 1 postsutural supra-alar; 1 postalar; 2 long and 2 much shorter scutellars; laterotergite with numerous setae. **Legs** (including coxae) brown, microtrichose, black setose. Fore femur with short anteroventral setae, posteroventral and posterior setae nearly as long as femur width. Fore tibia with setae dorsally (in apical part of tibia) nearly twice as long as tibia width (and similar setae present on dorsal side of tarsomeres 1–4 (so they are covered with setae longer than length of particular tarsomere), ventral setae short. Mid femur with anteroventral setae about as long as femur width, posteroventrals longer, in apical third nearly twice as long. Mid tibia and tarsus similarly setose and setose as fore tibia and tarsus, dorsal setae slightly longer and stronger than setae on fore leg and also ventral setae somewhat longer. Hind femur with row of 6–8 strong anteroventral spines in apical third (slightly shorter than femur width) and with several very long anterodorsal setae, otherwise short setose. Hind tibia (Figure 21B) peculiarly shaped, strongly curved ventrally on base, dorsally with several setae nearly twice as long as tibia width, ventral setae short (except several longer setae situated on incurved part); 1 very short seta in posteroapical comb. Basitarsus of all legs slightly swollen and long setose, hind basitarsus with rather strong setae dorsally. **Wing** clear, stigma brown, veins yellowish brown; CuA+CuP absent in apical part. Costal seta present, axillary excision 90º. Measurements: M_2_/dm = 1.3–1.4, M_4_ ap/mp = 1.8, wl/ww = 2.6. Halter brown, calypter brown with dark fringes. **Abdomen** brown, microtrichose, only extreme base of epandrium and small part of segment 8 shiny. Setae all dark. Chaetotaxy: posteromarginal setae on sides of tergite 2 slightly shorter than corresponding segment, on segment 3 about 1/3 as long, on segments 4–6 they very short, discal setae even shorter; dorsum of tergites short setose. **Terminalia** (Figure 21C, not dissected) very similar to *R. seticauda* sp. nov. Epandrial lamella with much finer setae ventrally and with similar comb of setae on apex. Cercus with peculiar tooth in dorsal view, “subcercal” process obviously absent.

**Female** (Figure 21D). Length: body 2.9–3.3 mm, wing 3.1–3.4 mm. Similar to male but with the following exceptions. Eyes broadly dichoptic, dorsalmost facets slightly smaller than ventral ones. Frons 0.12? mm broad and 0.15 mm long, with a few short setae on each side. Labrum 2/3 as long as head is high. Thorax exactly as in male. Legs including coxae similarly coloured as in male, however, differently setose. Fore femur short setose, anteroventral and posteroventral surface in basal half bare. Fore tibia short setose, without differentiated setae. Mid femur without anteroventrals on basal half and with short ones apically, dorsal setae and posteroventrals in apical third very slightly flattened, both slightly shorter than femur width. Mid tibia short setose. Hind femur with setation on both sides shorter than femur width and with a row of several strong and slightly flattened anteroventral setae in apical third nearly as long as femur width. Hind tibia slightly flattened and broadened in basal part (in the same place as curvature on male tibia), dorsal homogeneous and very slightly flattened setation is slightly shorter than tibia width, ventral setae shorter except several longer setae in the place of swelling. Basitarsi of all legs (including all remaining tarsomeres) slender, short setose. Wing dark brown, lighter in axillary area, stigma equally dark; measurements: M_2_/dm = 1.4–1.5, M_4_ ap/mp = 1.7–1.9, wl/ww = 2.1–2.2.

**Differential diagnosis:** *Rhamphomyia* (*P.*) *subcurvitibia* sp. nov. belongs to the species-rich group of *Pararhamphomyia* with uniserial dorsocentral setae, dark legs and entirely black setose body. This new species differs from any one of them (and also from similar species with pale legs) by dark halter and strongly curved hind tibia. The most allied species is undoubtedly *R. seticauda* sp. nov. (compare discussion under the later species). The female is similar to *R. minutissima* Saigusa and *R. omogoensis* Saigusa in having uniserial dorsocentrals, dark legs and halter, mid and hind femora with flattened setae and dark brown wing [25]. However, it differs from them in slightly broadened hind tibia near base and details of leg chaetotaxy.

**Distribution:** Russia (Primorskiy Territory).

**Dates of occurrence:** June.

### 3.22. Rhamphomyia (Pararhamphomyia) zeegersi sp. nov.

Zoobank link: urn:lsid:zoobank.org:act: 5E582045-FD6D-4789-9D8D-5EB9DB074A91

(Figure 22)

**Type material: HOLOTYPE** ♂, labelled: **Russia**: Altay + 2000 m Seminsky pass, Sarlyk mountain, valley, UTM: 45U 4085657 28.vi.2013, A. v. Eck (CULSP); **PARATYPES: RUSSIA, Altay Rep.:** 1700–1900 m, E of Seminskyi pass, 50°03′ N, 87°39′ E, 28.vi.2013, Th. Zeegers (3 ♂, 1 ♀, CULSP); Seminskiy pass, 1800 m, 51.05º N 85.62º E, 30.vi.2009, A. Barkalov (2 ♂, ZISP); Seminskiy Pass Hotel (RUS), 27–29.vi.2013, leg. M.P. van Zuijen MT (1 ♂, ZISP); Ulaganskiy District, Kurayskiy Ridge, 2500–2700 m, 50.33º N 87.75º E, tundra, 3.vii.2008, A. Barkalov (1 ♂, SZM); Ulaganskiy District, Kurayskiy Ridge, 2500–2800 m, 50.33º N 87.75º E, 29–30.vi.2008, A. Barkalov (1 ♂, SZM); Actru Glacier, 2–3.vii.2023, leg. A. van Eck & M.P. van Zuijen (1 ♂, 1 ♀, ZISP).

**Diagnosis:** Medium-sized (body about 4 mm) black and black setose species of *Pararhamphomyia* with multiserial dorsocentral setae, vein CuA+CuP incomplete and dark halter. Male: mid basitarsus straight and unmodified, hypopygium of *R. transversipyga* type; epandrium with short dorsoapical spines, phallus mostly thin, very long, broadly Z-like curved, with small loops in basal part. Female: legs without pennate setae, wings brownish.

**Etymology:** The species epithet, *zeegersi*, is a patronym in honour of Dr. Theo Zeegers (*Leiden, the Niderlands*), well-known Diptera specialist, collector of a part of the type series.

**Figure 22:**
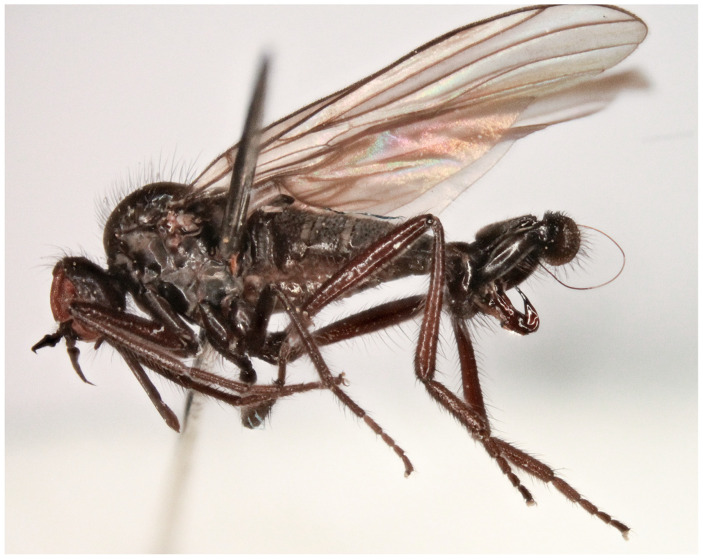
*Rhamphomyia* (*Pararhamphomyia*) *zeegersi* sp. nov., male habitus, lateral view.

**Description: Male** (Figure 22). Length: body 3.7–4.3 mm, wing 3.6–4 mm. **Head** regions black, brownish grey microtrichose (except noted), black setose. Eyes holoptic, upper ommatidia enlarged. Frons bare. Ocellar triangle with several equally fine setae, 2 anterior setae longer and nearly as long as postocular setae. Face broad, narrowly lustrous along oral margin, bare. Occiput covered with densely, long, uniform, fine setose (setae more than 0.30 mm long), postocular row incomplete, confined to dorsal half. Antenna black, scape and pedicel short setose, postpedicel rather narrow at base; antennomere ratio = 12:8:38–42:10–12. Labrum blackish brown, shiny, slightly longer than head height. Palpus brown, with several rather long setae (up to 0.20 mm long). Gena narrow, lustrous; clypeus lustrous. **Thorax** black, dark brownish grey microtrichose, mesoscutum in dorsal view subshiny, uniformly dark, without vittae. All setae black. Chaetotaxy: antepronotum with 4–5 short setae on each side; proepisternum with tuft of about 15 fine setae in lower part, bare on upper part; prosternum bare; postpronotal lobe with 1 longer seta barely differentiated from surrounding fine setae of different lengths (sometimes undistinguishable); acrostichal setae biserial, fine, about 15 in each row, long (up to 0.25 mm); dorsocentrals multiserial, on presutural area densely spreading laterally down to anterior part of notopleuron, subequally long as acrostichals, ending in several slightly longer setae reaching lateral parts of prescutellar depression; presutural intra-alar and presutural supra-alar setae not differentiated from nearby setation; notopleurals scarcely differentiated from similar setae on anterior part of notopleuron; supra-alar and prealar area with several hairs similar to other mesoscutal setae; 1 postalar; 6 scutellars; laterotergite with numerous setae. **Legs** blackish brown, microtrichose and black setose; coxae concolorous with pleura. Fore femur with row of fine, moderately long anteroventrals (as long as femur width) and longer posteroventrals (up to twice longer than femur width), posterior setae as long as femur width, short setose dorsally. Fore tibia with very short setulae ventrally and almost uniform, long, posterodorsal setae (at least twice longer than tibia width). Mid femur with somewhat longer setae close to base dorsally (about as long as femur width); anteroventral row irregular, with sparse, short setae (shorter than femur width, stronger setae close to base); posteroventral row interrupted, consisting of long setae on basal third of femur (longer than femur width) and short setae on apical third (nearly as long as femur width), middle third of femur without setae; mostly whitish tomentose ventrally. Mid tibia with row of long, spine-like, posteroventral (nearly 2× longer than tibia width) as well as shorter and finer anteroventral setae; 3–5 long anterodorsals (up to 3× longer than tibia width) with shorter and finer setae between them, similar fine setae also on posterodorsal part, strong posterodorsals not differentiated. Hind femur slightly narrowed and very slightly curved on basal part, with fine anteroventrals and posteroventrals up to 2× longer than femur width on apical third and shorter basally. Hind tibia very slightly dilated apically, ventral setae as long as tibia width about middle, shorter basally and apically; dorsally with uniform moderately long setae (up to 1.5× longer than tibia width); 1 long seta in posteroapical comb. Fore basitarsus with long setae posterodorsally (like those on fore tibia) and with stronger setae ventrally (longer than basitarsus width). Mid basitarsus with longer setae anteriorly and ventrally. Hind basitarsus short setose. Remaining tarsal segments on all legs without conspicuous features. **Wing** light brownish tinged, stigma slightly darker, veins brown, CuA+CuP incomplete. Costal seta poorly differentiated (2–3 setae slightly longer than nearby setae), axillary excision 90º or slightly obtuse. Measurements: M_2_/dm = 1.2–1.4, M_4_ ap/mp = 1.9–2.3, wl/ww = 2.9–3.2. Halter black, calypter black with black fringes. **Abdomen** black, in dorsal view densely very light grey microtrichose but in lateral view tergites become darker microtrichose; sternites light grey microtrichose but sternites 2–4 rather subshiny, remaining sternites densely microtrichose. Setae all black. Chaetotaxy: dorsum of abdomen very short setose (on segment 3 and following segments only about 0.04 mm long), lateral setae on segments 2–3 about as long as their corresponding segment, on remaining segments setae much shorter, posteromarginal setae not differentiated; sternites short setose except longer setae on sternite 2. Sternite 8 enlarged, densely covered with long setae. **Hypopygium** very large; epandrial lamella and cercus dark brown, subshiny, black setose. Epandrial lamella elongate, rounded apically, ventrally with long setae, dorsoapically with tuft of short spines. Hypandrium rim-like. Cercus long and low, straight dorsally, shorter than epandrial lamella, covered with fine setulae. Phallus very long, strongly sclerotised, mostly hair-like (thickened just beyond basal curvature), broadly Z-like curved, with small loops in basal part.

**Female.** Head dichoptic, ommatidia of subequal size. Frons 0.20 mm wide at middle, widening dorsally, with 2 rows of short setulae. Thoracic setae shorter than in male, acrostichals about 0.10 mm long and dorsocentrals slightly longer. Legs very short setose, all femora with ventral setae scarcely half as long as femora width, tibiae with setae shorter than their width. Wing darker brown than in male. Abdomen rather light brownish grey, short setose, lateral setae on segments 2–3 scarcely 1/3 as long as segments, on remaining segments even shorter; cercus long, slender.

**Differential diagnosis:** The species described above belongs to the *R.* (*Pararhamphomyia*) *transversipyga* group [3]. It differs from remaining species of this group by a combination of the following characters: mid basitarsus straight and unmodified, short dorsoapical spines on epandrium, phallus with small outgoing loop on basal part (hidden between epandrial lamellae), sternite 8 enlarged, and mid femur with anteroventral setae shorter than femur width. Female belongs to difficult group of species with legs without pennate setae, dark halter, incomplete anal vein and brownish wings. More studies are necessary to elucidate exact identification.

**Distribution:** Russia (Altay Republic).

**Dates of occurrence:** End of June–July.

## 4. Catalogue of Described Species

### 4.1. *Subgenus* Rhamphomyia *(*Calorhamphomyia *Saigusa)*

*Rhamphomyia* (*Calorhamphomyia*) *iridescens* sp. nov. Type locality: Russia, Altay Territory, Krasnoschekovskiy Distr., Tigirek vill., 51°08′ N, 83°03′ E, 550 m. Global distribution: Mongolia, Russia (East Siberia).

### 4.2. *Subgenus* Rhamphomyia *(*Pararhamphomyia *Frey)*

We follow the species groups of *Rhamphomyia* (*Pararhamphomyia*) proposed by Barták & Kubík [3], except noted.

### 4.3. “basitarsata” Species Group

*Rhamphomyia* (*Pararhamphomyia*) *basitarsata* sp. nov. Type locality: Russia, Primorskiy Terr., 42.7º N 131.1º E, Andreevka env. Global distribution: China, Russia (Far East).

**Remarks.**  The group is proposed herein to include a single species. See Differential Diagnosis for discussion.

### 4.4. “ciliatopoda” Species Group (Sensu Saigusa [25], Revised by Barták & Kubík [3])

*Rhamphomyia* (*Pararhamphomyia*) *sausai* sp. nov. Type locality: China, Jilin prov. Da Gu Jia 20 km SEE of Jilin 43.793º N, 126.78º E, 350 m. Global distribution: China.

### 4.5. “fuscipennis” Species Group

*Rhamphomyia* (*Pararhamphomyia*) *bifurcata* sp. nov. Type locality: Russia, Amurskaya Prov, town Zeja [53°44′ N 127°15′ E]. Global distribution: Russia (north of European part, East Siberia, Far East).

### 4.6. “norgensis” Species Group

*Rhamphomyia* (*Pararhamphomyia*) *norgensis* sp. nov. Type locality: Norway, Vågåmo [61°53′ N, 9°6′ E]. Global distribution: Norway, Russia (East Siberia, Far East).

**Remarks.**  The group is proposed herein to include *R. norgensis* sp. nov., *R. araneipes* Frey, *R. deformicauda* Saigusa and *R. wrangeli* Shamshev & Sinclair. See Differential Diagnosis under *R. norgensis* sp. nov. for discussion.

### 4.7. “parvula” Species Group

*Rhamphomyia* (*Pararhamphomyia*) *subcurvitibia* sp. nov. Type locality: Russia, Primorskiy Territory, Kamenushka [43°37′ N 132°13′ E]. Global distribution: Russia (Far East).

### 4.8. “praestans” Species Group

*Rhamphomyia* (*Pararhamphomyia*) *angustitibia* sp. nov. Type locality: Russia, Indigirka River, lower flow of Ystyn-Yuryakh River, Momskiy District [66°27′ N 143°13′ E]. Global distribution: Russia (East Siberia).

*Rhamphomyia* (*Pararhamphomyia*) *krivosheinae* sp. nov. Type locality: Russia, Amurskaya Prov., town Zeya [53°44′ N 127°15′ E]. Global distribution: Russia (East Siberia).

### 4.9. “pusilla” Species Group (Sensu Sinclair et al. [13])

*Rhamphomyia* (*Pararhamphomyia*) *indigirka* sp. nov. Type locality: Russia, Indigirka River, mouth of river Injali [65°14′ N 143°08′ E]. Global distribution: Russia (East Siberia).

*Rhamphomyia* (*Pararhamphomyia*) *morgunovka* sp. nov. Type locality: Turkmenistan, Serkhetli (35°17′ N 62°23′ E), env. Kushka. Global distribution: Turkmenistan.

### 4.10. “tibiella” Species Group

*Rhamphomyia* (*Pararhamphomyia*) *plutenkoi* sp. nov. Type locality: Russia, Primorskiy Terr., Artem [43°21′ N 132°11′ E]. Global distribution: Russia (Far East).

### 4.11. “tipularia” Species Group (Sensu Sinclair et al. [13])

*Rhamphomyia* (*Pararhamphomyia*) *amurensis* sp. nov. Type locality: Russia, Amurskaya Prov., town Zeja [53°44′ N 127°15′ E]. Global distribution: Russia (East Siberia).

*Rhamphomyia* (*Pararhamphomyia*) *epandriata* sp. nov. Type locality: Russia, Yakutia-Sakha, Indigirka River, mouth of river Injali [65°14′ N 143°08′ E]. Global distribution: Russia (East Siberia).

*Rhamphomyia* (*Pararhamphomyia*) *globulicauda* sp. nov. Type locality: Russia, Yakutia-Sakha, Indigirka River, lower flow of river Pystan-Yuryakh, Momskij District [66°27′ N 143°13′ E]. Global distribution: Russia (East Siberia, Far East).

*Rhamphomyia* (*Pararhamphomyia*) *nudifemorata* sp. nov. Type locality: Russia, Amurslaya Prov., town Zeja [53°44′ N 127°15′ E]. Global distribution: Russia (Far East).

### 4.12. “transversipyga” Species Group

*Rhamphomyia* (*Pararhamphomyia*) *zeegersi* sp. nov. Type locality: Russia, Altay, Seminsky pass, Sarlyk mountain valley, 2000 m. Global distribution: Russia (East Siberia).

### 4.13. *Species Unplaced to Groups*

*Rhamphomyia* (*Pararhamphomyia*) *acuticauda* sp. nov. Type locality: Slovakia, Kamenín, salty meadow, 49°53′ N, 18°39′ E, 130 m. Global distribution: Slovakia.

*Rhamphomyia* (*Pararhamphomyia*) *haladai* sp. nov. Type locality: Kazakhstan, Almaty Prov., 8 km W of Saryzhaz [42°54′ N 79°36′ E], 1900 m. Global distribution: Kazakhstan.

*Rhamphomyia* (*Pararhamphomyia*) *schachti* sp. nov. Type locality: Spain, Granada, Sierra Nevada, Puerto d.l. Ragua [37°06′ N 3°01′ E], 1700 m. Global distribution: Spain.

*Rhamphomyia* (*Pararhamphomyia*) *seticauda* sp. nov. Type locality: Russia, Primorskiy Terr., Kamenushka [43°37′ N 132°13′ E]. Global distribution: Russia (Far East).

*Rhamphomyia* (*Pararhamphomyia*) *spinicauda* sp. nov. Type locality: Russia, Amurskaya Prov., town Zeja [53°44′ N 127°15′ E]. Global distribution: Russia (Far East).

**Remarks.**  All species unplaced to groups belong to a large, heterogeneous assemblage of *Rhamphomyia* (*Pararhamphomyia*) members sharing uniserial dorsocentral setae. This part of the subgenus remains ungrouped.

## Data Availability

The original contributions presented in this study are included in the article. Further inquiries can be directed to the author.
